# Improved MALDI-MS Imaging of Polar and ^2^H‑Labeled Metabolites in Mouse Organ Tissues

**DOI:** 10.1021/acs.analchem.5c00620

**Published:** 2025-05-13

**Authors:** Siva Swapna Kasarla, Antonia Fecke, Karl William Smith, Vera Flocke, Ulrich Flögel, Prasad Phapale

**Affiliations:** 1 28371 Leibniz-Institut für Analytische Wissenschaften—ISAS—e.V., Otto-Hahn-Str. 6b, Dortmund 44227, Germany; 2 Experimental Cardiovascular Imaging, Institute for Molecular Cardiology, 9170 Heinrich Heine University Düsseldorf, Düsseldorf 40225, Germany; 3 Cardiovascular Research Institute Düsseldorf (CARID), Düsseldorf 40225, Germany; 4 Department of Environmental Science, Aarhus University, Frederiksborgvej 399, Roskilde 4000, Denmark

## Abstract

Imaging small polar metabolites and analyzing their *in vivo* dynamics with stable isotope-labeled (SIL) tracing through various biochemical pathways, including the citric acid (TCA) cycle, glycolysis, and amino acid metabolism, have gained substantial interest over the years. However, imaging these small polar metabolites across different tissue types is limited due to their lower ionization efficiencies and ion suppression from larger abundant biomolecules. These challenges can be further exacerbated with SIL studies, which require improvements in sample preparation and method sensitivity. Solvent pretreatments before matrix application on a tissue section have the potential to improve the sensitivity of metabolite imaging; however, they are not yet widely optimized across tissue types. Furthermore, there is a recurring concern about metabolite delocalization from such wash treatments that require “spatial validation”. Here, we optimized a simple “basic hexane” wash method that improved sensitivity up to several folds for a broad range of polar and ^2^H-labeled metabolites across five different mouse organ tissues (kidney, heart, brain, liver, and brown adipose tissue). Notably, we provided region-specific quantification of 51 metabolites using laser microdissection (LMD)-LC-MS/MS to validate their localization observed in MALDI-MSI analysis after the basic hexane wash. Overall, we reported an improved MALDI-MSI sample pretreatment method with a “spatial validation” workflow for sensitive and robust imaging of polar metabolite distributions in mouse organs.

## Graphical abstract

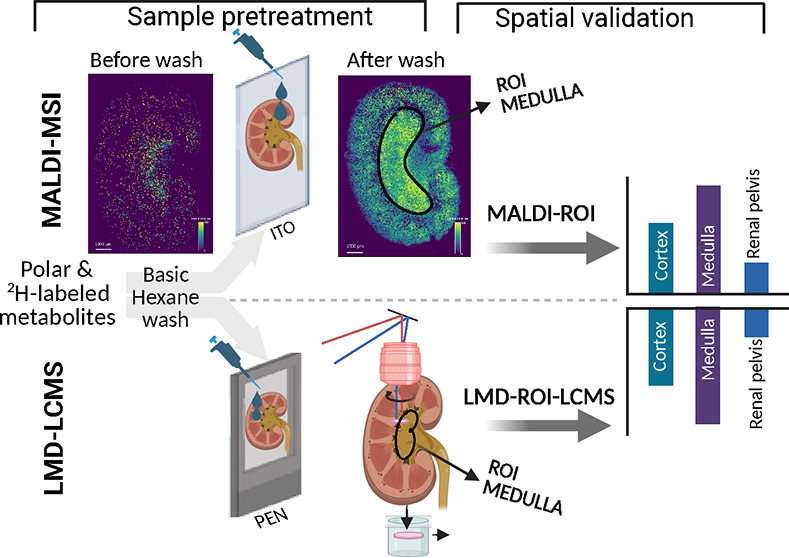

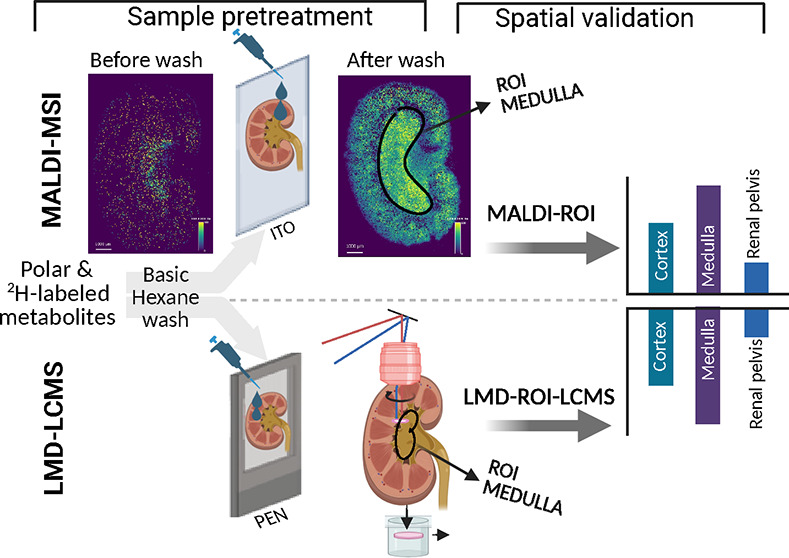

## Introduction

Matrix-assisted laser desorption/ionization mass spectrometry imaging (MALDI-MSI) is a label-free molecular technique for visualizing the spatial distribution of molecules (metabolites, lipids, peptides, xenobiotics, and drugs) in tissue complementing traditional histopathology.^1^ Unlike bulk tissue analysis (e.g., LC or GC-MS), MSI provides spatially resolved analysis to decipher metabolic heterogeneity at near cellular resolution.^2–4^ MALDI-MSI is rapidly becoming the leading technology due to its better molecular coverage and lateral resolution compared to techniques such as LDI and DESI.^5,6^ However, small polar metabolites (*m*/*z* < 500) still suffer from poor sensitivity and coverage.^7^ This can be largely attributed to their poor MS ionization efficiencies and suppression of ion signals from competing molecules (e.g., lipids and proteins) in tissues. In particular, polar metabolites of glycolysis, TCA cycle, and amino acid pathways are of greater interest in understanding the pathophysiology of cancer and other metabolic diseases.^1^ MALDI-MSI methods to detect these polar metabolites are not widely accessible compared to the imaging of lipids, peptides, and drugs.^7,8^ Furthermore, these metabolites are water-soluble and labile in nature, which can cause their delocalization from native tissue locations during sample treatments.

Recent developments in spatial metabolomics made a systematic improvement in metabolite imaging using new matrices, sample pretreatment strategies, and optimized MALDI instrumentation parameters.^9,10^ For example, the (1-naphthyl)­ethylenediamine dihydrochloride (NEDC) matrix^11^ and tissue chemical derivatization (OTCD) methods^12,13^ have shown promising results for small molecules and are now being widely adopted for metabolite imaging. Sample pretreatment strategies including “washing off” the undesired biomolecules have considerably reduced ion suppression effects and improved the sensitivity of targeted metabolite classes and drugs.^14,15^ But these methods^16^ are still limited to a specific class of molecules and tissue types, for example, immersion in acetone for carnitines and cholines in a murine tumor model with osteosarcoma,^14^ ethanol treatment for carnitine metabolism in breast cancer tissues,^17^ chloroform wash of rat brain sections to improve the detection of molecules of *m*/*z* 150–500 in positive ionization mode,^15^ acidic methanol wash to improve the sensitivity of phosphate-containing energy metabolites,^10^ and hexane and trifluoroacetic acid wash for drug molecules.^18^ However, a comprehensive method covering major metabolic pathways has not yet been developed in different organ tissue types. Moreover, such methods have not been spatially validated for metabolite delocalization after wash treatment.

In recent years, laser capture microdissection (LMD) has gained substantial interest due to its capability for the precise excision of regions of interest (ROIs) within the tissue, which has been broadly studied with deep visual proteomics methods.^19^ However, combining MALDI-MSI with metabolomics workflows^20^ remains largely unexplored particularly for small metabolites (*m*/*z* < 500). Our spatial validation approach using LMD-LC-MS/MS-based metabolomics can be a powerful means to validate metabolite spatial distributions obtained from MSI data sets.

Further advances in MSI of stable isotope-labeled metabolites (SIL) allowed the tracking of metabolic pathway activities and estimation of their spatial fluxes in organ tissues and tumor ecosystems.^3,21^ However, the method for imaging ^2^H-labeled metabolites in tissues after infusion of ^2^H_7_-glucose in mouse models has not yet been reported due to the complexity of *in vivo* sampling, metabolome coverage, and detection of extremely low-abundance isotopologues.^21^ The optimized conditions for one tissue type may not be transferable to other tissue types due to differences in tissue structures and cellular heterogeneity. In this study, we have systematically optimized the solvent additive pretreatment conditions (basic hexane wash) to improve the ionization, sensitivity, and coverage of polar and ^2^H-labeled metabolites across five organs post ^2^H_7_-glucose infusion. Additionally, we performed region-specific quantification of selected metabolites using LMD-LC-MS/MS workflow on consecutive tissue sections to spatially validate MALDI-MSI results and assess metabolite delocalization.

## Methods

The details of materials, animal experiments, sample preparation, data acquisition parameters, and data analysis performed are detailed in the Supplementary Methods file.

### Solvent Treatments and Tissue Sample Preparation

To prepare the basic hexane wash solution, 28% aqueous ammonia solution was premixed (1 μL; 0.1%) with the cosolvent chloroform (2 μL; 0.2%) before adding it to hexane (997 μL). The mixture was used immediately to minimize the potential phase separation. This step improves the distribution of the basic modifier in the organic solvent and ensures consistency in the washing process.

Thaw-mounted 12 μm thick tissue sections were desiccated under a vacuum for 20 min followed by organic solvent wash treatment before matrix application. The “pipetting” wash method was adapted from the existing literature.^18^ Briefly, 100 μL of a freshly prepared washing solvent was placed on tissue sections for 5 s, and the slide was inclined to remove the solvent. The procedure was repeated five times with an overall 500 μL solvent for the removal of unwanted analytes and to ensure even exposure of the solvent on the tissue section. After solvent exposure, the tissue slide was desiccated again under a vacuum for 20 min followed by application of the freshly prepared NEDC matrix (7 mg/mL in 70:25:5 of MeOH/ACN/H2O (v/v/v)) using the SunCollect MALDI sprayer^2^ (for a detailed description, see Supplementary Methods).

### MALDI-MS Imaging

MALDI-MSI experiments were performed on an Orbitrap Q-Exactive HF mass spectrometer (Thermo Fisher Scientific GmbH, Bremen, Germany) coupled to an elevated-pressure MALDI (EP-MALDI) ion source (Spectroglyph LLC, Kennewick, WA, USA). The 349 nm Nd:YLF solid-state laser (Explorer One, Spectra Physics, Mountain View, CA) was operated at a repetition rate of 500 Hz and pulse energy of 1–2 μJ. The laser was focused on a spot size of 20 μm, and the pixel sizes for the labeled and unlabeled tissues were set at 40 μm. The mass spectrometer was operated in negative- and positive-ion mode in the mass range of *m*/*z* 50–550 ^22^ (for a detailed description of MALDI funnel operating parameters, see the Supplementary Methods).

### Laser Capture Microdissection (LMD)-LC-MS/MS-Based Metabolomics

The consecutive liver and kidney tissue sections (*n* = 4) were thaw-mounted on polyethylene naphthalate (PEN) membrane slides at −20 °C and stored at −80 °C until further use. The basic hexane wash strategy was applied to the tissues mounted on PEN membrane slides for LMD. Approximately 0.8 mm^2^ of liver tissue sections was excised using an LMD6000 (Leica, Wetzlar, Germany) to optimize the sample preparation and acquisition of LC-MS/MS-based metabolomics. Later, specific regions such as the cortex, medulla, and renal pelvis of the kidney tissue were excised (area ∼0.8 mm^2^) to perform spatial validation of specific metabolites. LC-MS/MS metabolomic analysis for the LMD excised tissues was performed by the optimized sample preparation method and in-house quality controls^23^ (see the Supplementary Methods file for a detailed explanation).

### Data Analysis and Metabolite Annotation

Preliminary images were evaluated in the ImageInsight (Spectroglyph LLC, USA) software, and MSI data processing was further performed with LipostarMSI v.2.1 (Molecular Horizons). Thermo RAW (.raw) and positional files (.xml) were converted to imzML files using the built-in converter for LipostarMSI, which utilizes MSconvert from ProteoWizard (3.0.22317)^24^ for the initial conversion to imzML format. The imzML file was then loaded into LipostarMSI, and the MALDI MS imaging data metabolites were manually selected and annotated using the LipostarMSI software.^25^ Metabolite annotations was guided by the METASPACE web application with 10% FDR within ±3 ppm mass accuracy and confirmed using LC-MS/MS, the in-house database,^26^ and on-tissue MS/MS for selected metabolites.

### Comparison between MALDI-MSI and LMD-LC-MS/MS

The metabolite intensities of the defined ROIs from the MALDI-MSI and LMD-LC-MS/MS were extracted from ShinyCardinal v3.4 and Progenesis QI, respectively. The statistical analysis of the metabolite intensities acquired from both the techniques was carried out in Metaboanlyst v6.0 separately. The heatmaps of the differential metabolites were constructed based on the ANOVA *p* value of <0.05. The bar graphs were obtained from the volcano plot analysis using a fold change >1.5 and *p* value of <0.05 to find the significant features.

## Results and Discussion

### Optimization of Solvent Treatment, MALDI-MSI, and Spatial Validation Workflow

This study provides an optimized workflow for improving the sensitivity and coverage of polar metabolite imaging. First, healthy tissues (kidney, liver, brain, heart, and brown adipose tissues) were isolated from a mouse infused with unlabeled or ^2^H_7_-labeled glucose for 30 min and flash frozen immediately in liquid nitrogen (Figure 1a). After cryosection of fresh frozen tissues, the optimized “basic hexane” wash method was applied to all tissue types, and data were acquired using a modified EP-MALDI-MSI setup for the lower mass range (Figure 1b).^22^ MS image analysis was performed using the LipostarMSI software based on METASPACE annotations with 10% FDR scores^27^ and confirmed with LC-MS/MS and on-tissue MS/MS analyses (Figure 1c).^28^ Metabolite signal intensities were extracted to assess the impact of the studied wash solvent conditions on the respective tissue sections. Exemplary images of unlabeled and ^2^H-labeled metabolites showing improved sensitivity after basic hexane wash are shown for consecutive mouse kidney sections (Figure 1d). Subsequently, spatial validation was performed by serial sectioning of kidney tissues on a glass slide for H&E staining, PEN membrane slide for LMD-LC-MS/MS analysis, and indium tin oxide (ITO) slide for MALDI-MSI analysis. The validation of metabolite localization was performed with MALDI-ROI analysis and region-specific LMD-LC-MS/MS metabolomics of the excised tissue regions (renal pelvis, medulla, and cortex; Figure 1e).

**Figure 1:**
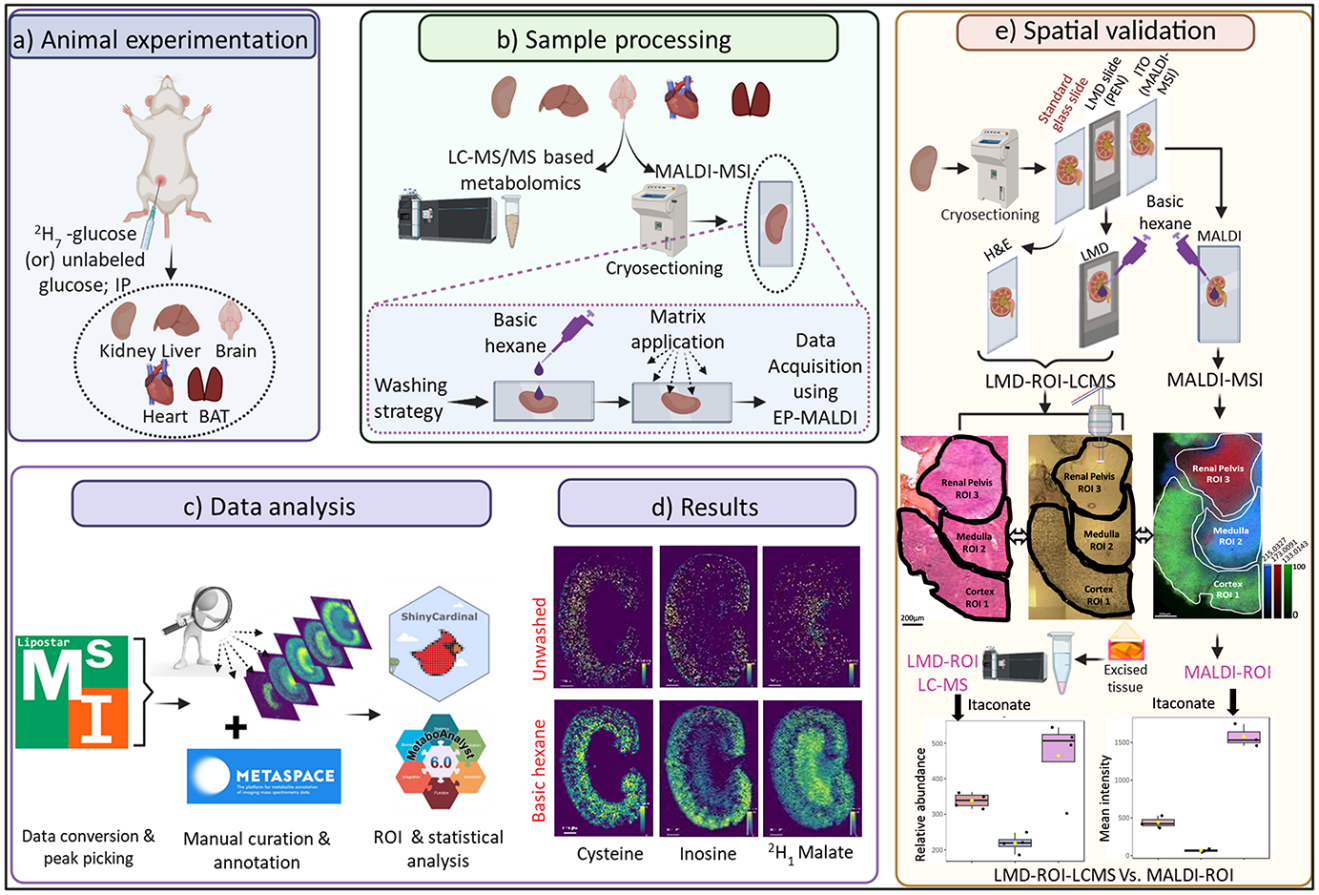
Optimized workflow for polar metabolite imaging using MALDI-MSI and spatial validation with LMD-LC-MS/MS. (a) Animal experimentation: unlabeled glucose (*n* = 3) and ^2^H_7_-glucose (*n* = 3) infused in mice via intraperitoneal injection (IP) for about 30 min followed by organ collection. (b) Tissue sample processing: Organs were further processed for LC-MS/MS and MALDI-MSI experiments. Before application of the NEDC matrix, the samples underwent “basic hexane” washing treatments followed by data acquisition using the optimized EP-MALDI-MSI method. (c) Data analysis was performed using the LipostarMSI software, and peaks were annotated with accurate ^m^ass (±3 ppm) and METASPACE FDR scores and further confirmed with reference standards or LC-MS/MS analysis of consecutive tissue sections. (d) Basic hexane wash: Selected MALDI-MS ion images of metabolites (*m*/*z* 131.0462; cysteine, [M – H]^−^ *m*/*z* 120.0124; inosine, [M – H]^−^ *m*/*z* 267.0735; and ^2^H_1_-malate, [M – H]^−^ *m*/*z* 134.0205) depict the improved signal intensity after the basic hexane wash. Scale bar: 1 mm. Pixel size: 40 μm. (e) Spatial validation workflow: H&E staining, LMD-LC-MS/MS metabolomics, and MALDI-MSI were performed to validate region-specific metabolite localization for MALDI-ROIs from the renal pelvis, medulla, and cortex (images created with the BioRender scientific illustration software).

### Basic Hexane Wash Improves the Sensitivity of Polar Metabolites

The sensitivity of the polar metabolites using MALDI-MSI can be enhanced by reducing the ion suppression from competing abundant molecules such as lipids and by improving ionization efficiencies of these metabolites. To achieve this, we systematically investigated the effect of commonly reported^10,15^ organic solvents chloroform and hexane in removing the lipid content of the tissue membrane. We tested five different tissues (kidney, liver, brain, heart, and BAT) with 500 μL of both solvents as described in the Methods section. We found that the hexane and chloroform wash showed similar metabolite coverage but variable signal intensities between tissue types. For example, the chloroform wash showed a slight improvement in the coverage of metabolites, identifying 151 metabolites in the kidney and 80 in the brain compared to the hexane wash identifying 148 in the kidney and 78 in the brain (Figure 2a,b and Table S1). On the other hand, the hexane wash improved metabolite coverage with 94 metabolites in the liver, 106 in BAT, and 89 in the heart compared to the chloroform wash (83 metabolites in the liver, 98 in BAT, and 81 in the heart). However, in terms of metabolite signal intensities, certain metabolites such as inosine, acetyl-glycine, ornithine, etc. (Figure 2c), showed a higher fold change with the hexane wash compared to chloroform. The solvents collected after the tissue wash were mixed with a matrix (sample-to-matrix ratio 1:3) and then analyzed with similar MALDI-MS parameters. In the collected wash solution, we found the removal of several lipid classes with both the hexane and chloroform wash (Figures S1 and S2), which details the process of delipidation. Furthermore, we also investigated the two mixtures of hexane and chloroform in a ratio of 70:30 (relatively nonpolar) and 30:70 (relatively polar) to evaluate their effect on improving sensitivity and metabolite coverage in the liver (Figure S3). The average spectra of the combination of collected hexane and chloroform wash solvent (70:30) show increased detection of lipid ion masses in a high mass range (*m*/*z* 350–1200). This demonstrates the efficiency of hexane over chloroform in the removal of higher-abundance lipids from a broader mass range. Additionally, the spectra for the 30:70 (hexane/chloroform) ratio showed comparatively fewer *m*/*z* peaks (Figure S3), underscoring the effectiveness of chloroform (nonpolar solvent). This supports the use of hexane (highly nonpolar solvent) over chloroform wash to improve the detection of polar metabolites. Therefore, we selected hexane as a primary solvent for further optimization with additives.

**Figure 2:**
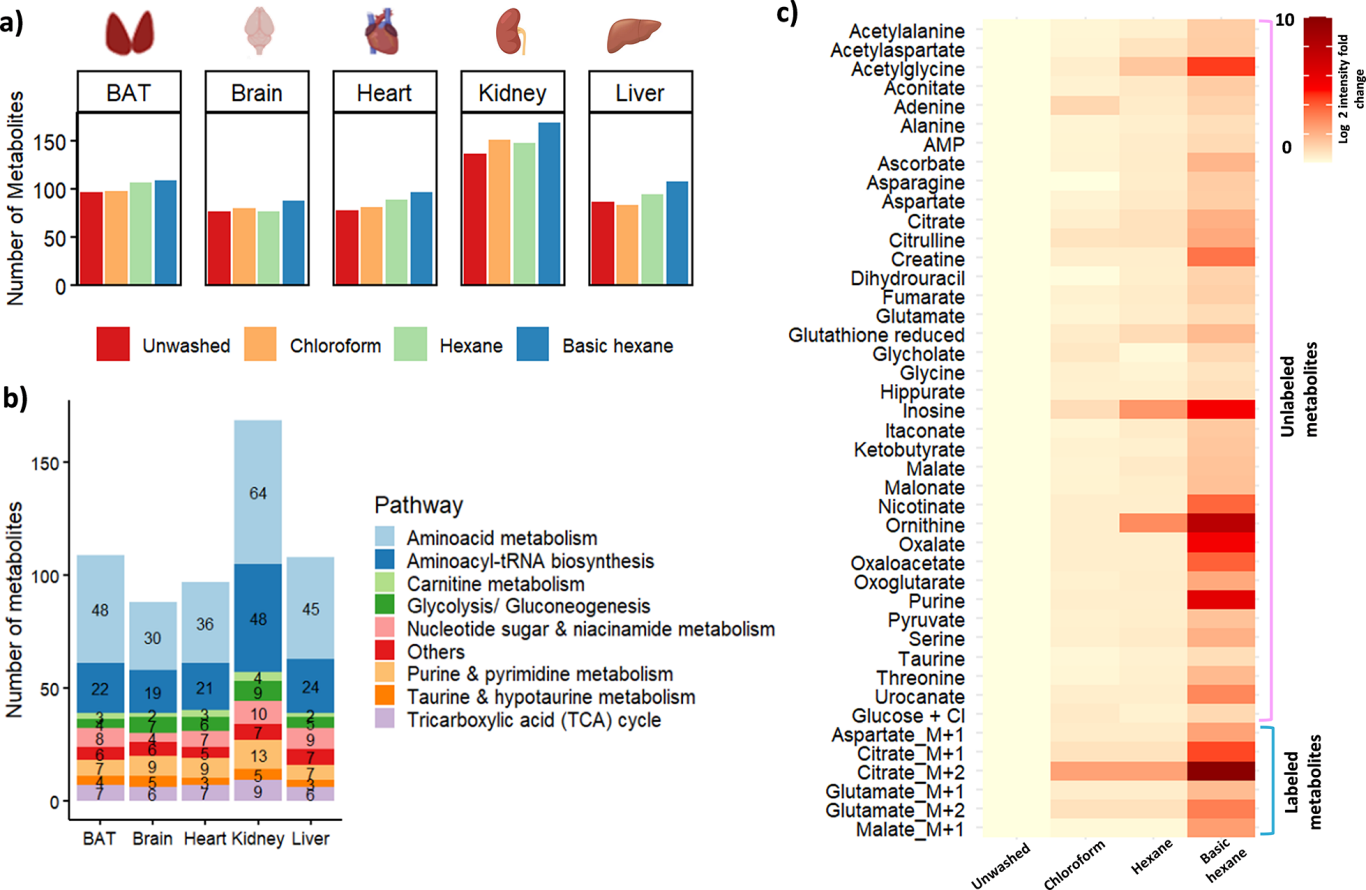
Improved metabolite detection across five tissue types after basic hexane wash. (a) Comparative bar graphs depicting the number of metabolites identified from EP-MALDI-MSI analysis after different wash conditions for five tissues. (b) Metabolites identified with basic hexane wash cover key metabolic pathways (KEGG database) in all tissue types. (c) Heatmap showing the improvement in the scaled fold change of log2 signal intensities of most metabolites and ^2^H-labeled isotopologues after the basic hexane wash compared to other solvents relative to unwashed tissue.

We then investigated the addition of basic additives to improve the ionization efficiency of polar metabolites in the negative ionization mode. Since most of the polar metabolites tend to deprotonate in the negative ion mode, we chose ammonium hydroxide (NH_4_OH) or aqueous ammonia, which is a common additive in hydrophilic interaction liquid chromatography (HILIC) LC-MS metabolomic analysis.^26,28,29^ Different concentrations of NH_4_OH were tested, i.e., 0.05, 0.1, and 1% NH_4_OH. Hexane with 0.1% aqueous ammonia under basic conditions was shown to improve the detection of polar metabolites such as glutamate, fumarate, aspartate, and itaconate (Figure S4). As solvent volume might tend to solubilize polar metabolites (counter effect) or dissolve proteins (synergistic effect), we tested exposure to 0.5, 1, and 2 mL volumes and collected wash for MS analysis to confirm the wash-off effect for lipids and polar metabolites. We found that 0.5 mL significantly improved the sensitivity of polar metabolites, thereby revealing a clearer, more uniform distribution of metabolites, and efficiently removed all the major lipid classes including LPC, LPE, PC, PE, and TG without any effect on polar metabolites. However, no significant differences were observed with increasing solvent volumes, which might be due to the saturation of membrane lipids (Figure S5). Furthermore, basic washing conditions might facilitate protein solubility and removal,^30^ which could reduce the ion suppression effects for metabolites. To investigate the latter, we collected tissue postwash to measure the protein content removed by basic hexane and found no significant amount of protein washed off (Table S2a). Therefore, the enhanced metabolite sensitivity can be attributed to the effective removal of lipids by hexane, as well as the improved ionization of metabolites facilitated by NH_4_OH under basic conditions. To further understand the superiority of basic conditions by aqueous ammonia in MALDI-MSI, we tested selective standards (pyruvate, serine, fumarate, aspartate, and hexose) mixed with only the NEDC matrix, NEDC + 0.1% aqueous ammonia, and NEDC + 0.1% formic acid (FA). Our results showed that the addition of basic modifiers has significantly improved the signal intensities of all studied metabolites (Figure S6). Later, the effect of basic additive in both the washing step and spraying step was also investigated. First, the prepared standard mixture was spotted without any treatment and addition of basic additive, i.e., unwashed standard spot sprayed with NEDC matrix solution (condition 1). Second, we tested whether ammonia directly affects ionization without washing, which helps to clarify if the modifier interacts with the matrix or metabolites, i.e., unwashed standard spot sprayed with NEDC + 0.1% aqueous ammonia (condition 2). The last one was the basic hexane wash alone, which addresses both the loss of metabolite due to washing as well as the surface interaction of the metabolite spotted with the basic additive, i.e., standard spot washed with basic hexane and sprayed with NEDC (condition 3). Figure S7 shows that condition 3 outperforms the other conditions by showing a significant improvement in the ionization efficiency of several metabolites, such as pyruvate, fumarate, malate, aspartate, and citrate, compared to the other studied conditions. These results support our hypothesis that the basic additive enhances ionization efficiency by altering the analyte’s surface properties and increasing the abundance of deprotonated ions.

The basic hexane wash strategy (hexane + 0.1% aqueous ammonia) markedly improved the ionization efficiency of polar acid metabolites compared to chloroform and hexane in all the measured tissues (Figure S8 and Table S1). The microscopic images of tissues stained with H&E before and after wash did not show any major alterations in tissue morphology (Figure S9). However, H&E of such fresh frozen tissues may not show cellular structures and nuclei. Hence, further complementary imaging may be necessary to provide a detailed assessment of cellular integrity. Specifically, this method allowed for the detection of 169 metabolites in the kidney, 109 in BAT, 108 in the liver, 97 in the heart, and 88 in the brain (Figure 2a, Tables S1 and S3–S7) covering key metabolic pathways, including glycolysis, TCA cycle, amino acids, purine metabolism, etc. (Figure 2b). Most of these metabolites also showed remarkable improvements in the signal intensity after the basic hexane wash, as shown in the kidney (Figure 2c), compared to other published protocols.^10,14,15^ The heat map of other tissues (Figures S10–S14) showed similar sensitivity improvement. (The list of metabolites with their relative signal intensities is provided in Tables S3–S7. The comparison of raw intensities from METASPACE and normalized intensities from the LipostarMSI software is provided Table S8 and Figure S15, which show excellent correlation (*R* ^2^ > 0.9).)

### Analytical Validation

The specific improvement observed in the sensitivity of highly polar small metabolites in the low *m*/*z* region, such as pyruvate, exhibits a ∼3.5-fold increase in the signal-to-noise ratio (S/N) (Figure 3a). Moreover, to ensure the reproducibility of our basic hexane wash method, we analyzed the three consecutive sections on three different days. The average intensities of the signal were used to calculate the relative standard deviation between replicates (*n* = 3). Representative images of acetylaspartate, citrulline, and inosine in the kidney section showed an acceptable relative standard deviation (RSD < 20%) while maintaining a consistent distribution of metabolites across replicates after the basic hexane wash (Figure 3b). Most of the metabolites' RSD values were below 20% (Table S9). Additionally, we performed bulk LC-MS/MS^28^ and on-tissue MALDI-MS/MS of matching tissue sections (Table S10) to confirm metabolite identifications. We also evaluated the performance of our method in positive ionization mode (2,5-dihydroxy benzoic acid; DHB matrix) and found a better coverage of the metabolites in negative ionization with the NEDC matrix (Figure S16). In addition, we applied our method to ^2^H-labeled tissues in negative ionization mode and found reproducible results (Tables S3–S7). Our method also demonstrates specificity and high spectral resolution, enabling the separation between natural ^13^C isotopes from ^2^H isotopologues for all metabolites (Figure S17).

**Figure 3:**
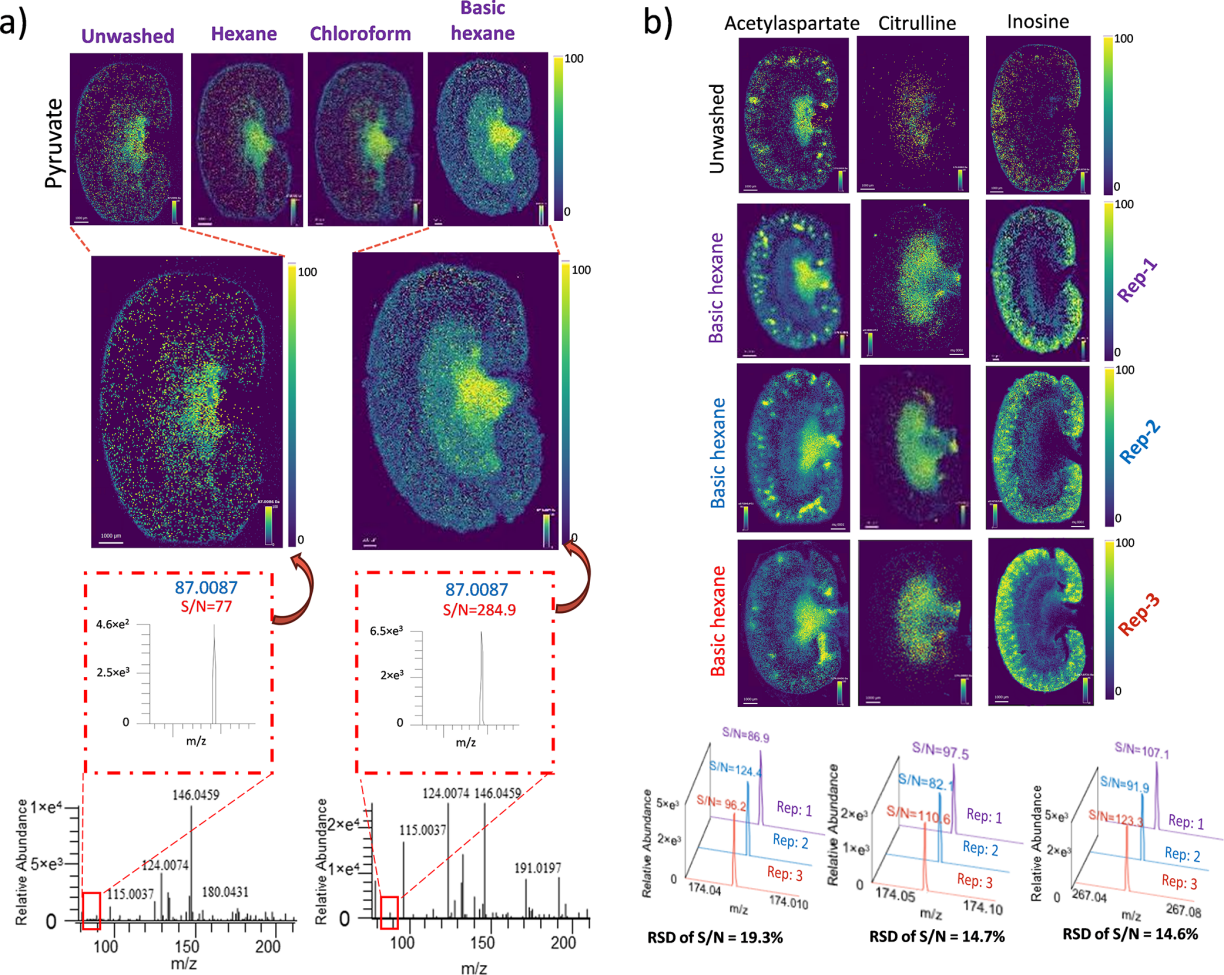
The basic hexane wash improves the sensitivity and reproducibility of selected metabolites in kidney tissue sections. (a) Effect of different wash conditions showing >3-fold improvement in sensitivity (S/N ratio) in the ion image of pyruvate after basic hexane wash in kidney sections acquired at 40 μm pixel size. (b) Repeat analysis of consecutive kidney tissue sections (*n* = 3) for acetylaspartate, citrulline, and inosine showed intensity variation with an acceptable range (RSD < 20%). Scale bar: 1 mm. RSD values for other metabolites are listed in Table S10.

### Spatial Validation of Metabolite Localization Using LMD-LC-MS/MS

LMD is a powerful tool for the precise isolation of specific cell populations or ROIs from tissue sections. The approach of combining MALDI-MSI and LMD has potential applications to understand the molecular heterogeneity within the tissue environment. It has been successfully applied to study the spatial molecular makeup of proteins, lipids, and peptides within tissue sections.^19^ Despite its potential, it has not been extensively applied to study small metabolites due to challenges in the sample preparation. Here we optimized the sample preparation process for low-volume LMD-LC-MS/MS analysis. To assess the effectiveness of the sample pretreatment strategy in enhancing metabolite sensitivity, we used liver tissues. The tissues mounted on PEN membrane slides were washed with basic hexane, and approximately ∼0.8 mm^2^ was excised using LMD. The collected low-input samples were used to tune the solvent for the extraction and reconstitution protocol for LC-MS/MS-based metabolomics analysis in negative ion mode (Figure S18). Overall, 94 metabolites were identified in negative ion mode, of which 68 metabolites showed improved signal intensity fold change greater than 2 (Table S11). This further complements the potential use of the basic hexane wash strategy to improve the ionization efficiency of polar metabolites regardless of ionization techniques such as ESI (Figure S18), which can be broadly effective across common MALDI sources and MS types.

Using this optimized LMD-LC-MS/MS-based method for kidney tissues, we addressed a critical concern in MSI regarding polar metabolite delocalization during sample treatments. The serial sections of the kidney tissue were mounted on the glass slide for H&E staining, ITO slide for MALDI-MSI, and PEN membrane slide for LMD-LC-MS/MS analysis. The spatial validation of selected metabolites was performed in the following steps: first, the MALDI-ROIs of the kidney were segmented by overlaying the selective metabolite ion images specific to the cortex ([malate – H]^−^; *m*/*z* 133.0143), medulla ([glucose + Cl]^−^; *m*/*z* 215.0327), and renal pelvis ([itaconate – H]^−^; *m*/*z* 173.0091), which showed excellent correlation with H&E-stained images of the kidney (Figure 4a–c). Second, the metabolite intensities from the MALDI-ROIs (Figure 4b) were extracted for quantitative comparisons with LMD-LC-MS/MS data. For this purpose, consecutive PEN membrane kidney tissue slides, treated with basic hexane, were overlaid with H&E and MALDI MS ion images. Similar ROIs were excised using LMD (Figure 4b). The excised low-input samples (*n* = 4) were then processed for LC-MS/MS-based metabolomics analysis to validate region-specific metabolite abundances, enabling direct comparison with MALDI-ROIs (Figure 4d).

**Figure 4:**
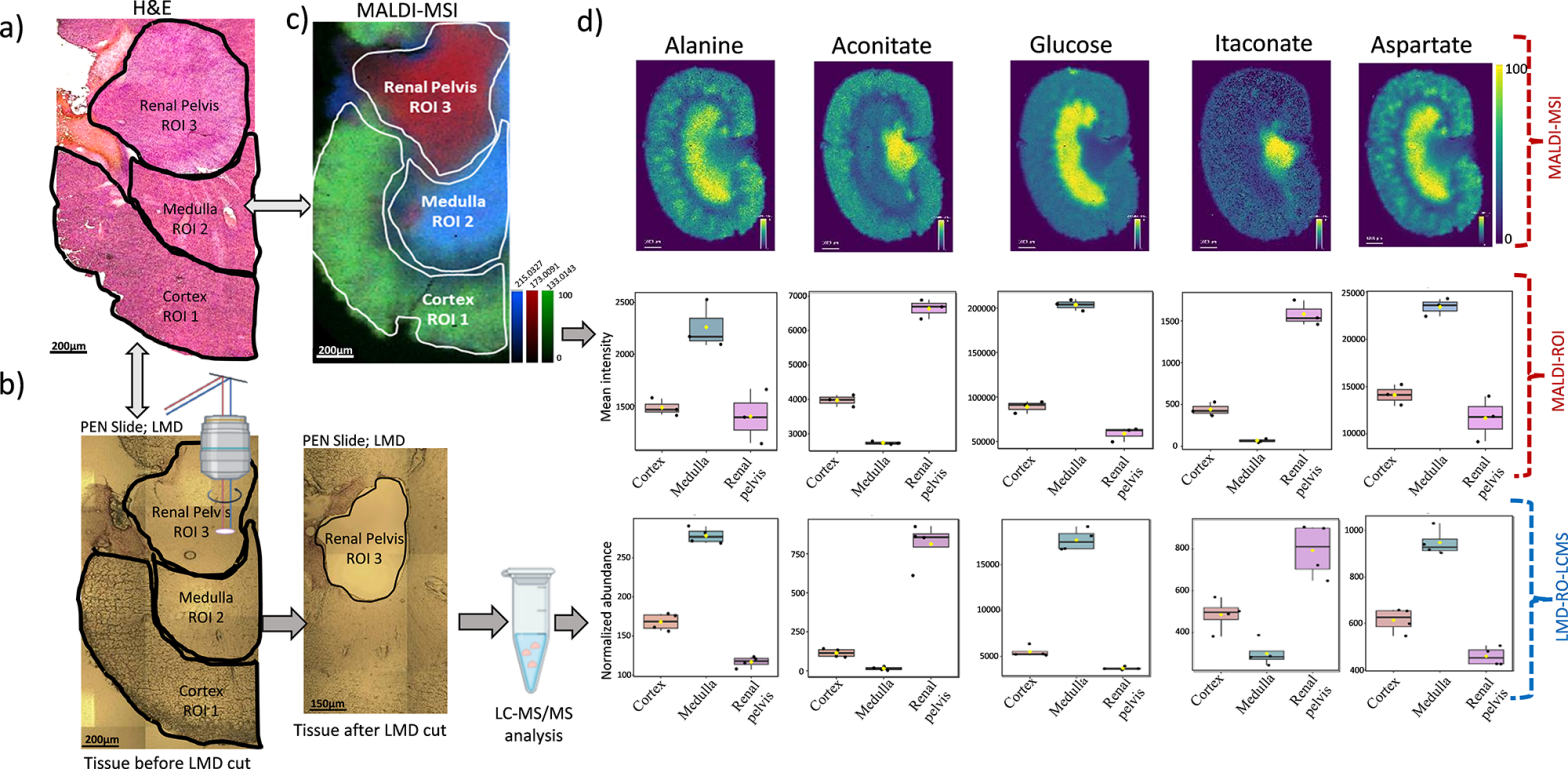
Spatial validation workflow in the kidney tissue after wash. (a) H&E-stained image of the kidney after basic hexane wash. (b) Defined ROIs of the basic hexane washed PEN membrane slide, which are consistent with H&E-stained images of consecutive sections (tissue before cut) and those excised using LMD (tissue after cut). (c) Overlaid ion images of glucose, malate, and itaconate after MSI analysis of post basic hexane wash kidney tissue. (d) MS ion images (scale bar: 1 mm) and boxplots of normalized intensities showing corroborated results from MALDI-ROIs and region-specific LMD-LC-MS/MS analysis. Boxplots for the remaining 51 metabolites are provided in Figure S22.

We identified 57 common metabolites between MALDI-MSI and LMD-LC-MS/MS analyses for all ROIs (Table S12), of which 51 metabolites showed quantitatively similar localization within the cortex, medulla, and renal pelvis (Figures S21 and S22). In the PCA plot, both MALDI and LC-MS data sets showed similar clustering patterns among the three kidney regions studied (Figure S19). For instance, glucose and alanine showed higher abundance in the renal pelvis followed by the cortex and medulla in both MALDI-MSI and LMD-LC-MS/MS analysis, which confirm the spatial localization of those metabolites (Figure 4d). Also, aconitate and itaconate showed relatively similar spatial distribution from both analytical techniques with high abundance in the renal pelvis followed by the cortex and medulla. Similarly, 51 metabolites showed a similar trend collectively in both MALDI-MSI and LMD-LC-MS/MS (Figure S20), which support that there was no metabolite delocalization after the basic hexane wash of the tissue sections.

### Improved SIL Metabolite Imaging

Stable isotope labeling (SIL) has recently emerged as a powerful strategy for studying nutrient metabolism in mammals.^2,3,21^ However, the sensitive detection of respective isotopologues from tissue samples is limited due to its labeling efficiency, low abundance, and ion suppression effects. There are several MALDI-MSI methods to detect ^13^C-labeled metabolites, but there are no methods that have been reported to image ^2^H_7_-labeled metabolites post ^2^H_7_-glucose infusion in a mouse model. Although it is less common, ^2^H-MSI offers complementary information to ^13^C labeling for tracing specific metabolic pathways and has a potential value in multimodal imaging with other modalities such as deuterium MRI or NMR.^31,32^ ^2^H_7_-glucose can provide position-specific targeted tracing of glycolysis and TCA cycle metabolic pathways. Therefore, we applied and evaluated our washing method to improve the detection of deuterated metabolites in these five mouse organ tissues post ^2^H_7_-glucose infusion. We found an increase in the intensity of the signal of the ^2^H-labeled metabolites in all tissues (Figure 5a, Figures S21–S24). For the first time, we report imaging of 10 deuterated metabolites with their isotopologues showing significant improvement in sensitivity of 8- to 10-fold among all the tissues studied. These metabolites cover three major metabolic pathways, namely, glycolysis, TCA cycle, and amino acid metabolism (Figure 5b). A distinct region-specific metabolism was observed in the kidney as it has highly specialized and compartmentalized metabolic functions. For example, ^2^H_7_-labeled glucose is localized more in the cortex region of the kidney, which is in agreement with the recent results showing higher localization of ^13^C_6_-glucose in the cortex.^33^ The differences could be due to the cortex having different choices of energy source (e.g., fatty acids) leading to less glucose respiration, which consequently localizes the labeled glucose in the cortex region. Conversely, the medulla is heavily dependent on glucose, resulting in the rapid metabolic conversion of glucose that reduces the localization of labeled metabolites in the medulla.^34,35^ Our results show similar minimal differences in the isotopic pattern of glucose across other studied tissues such as the brain, heart, liver, and BAT. Nevertheless, significant improvements in the sensitivity of labeled metabolites were observed in all tissue types, which can be potentially adapted to study metabolic differences in disease models such as cancer, ischemic injury, or metabolic disorders. This demonstrates the broader application of our method for studying *in vivo* mammalian metabolism and potentially other nutrient tracers that can enhance our understanding of metabolic regulation and dysfunction.

**Figure 5:**
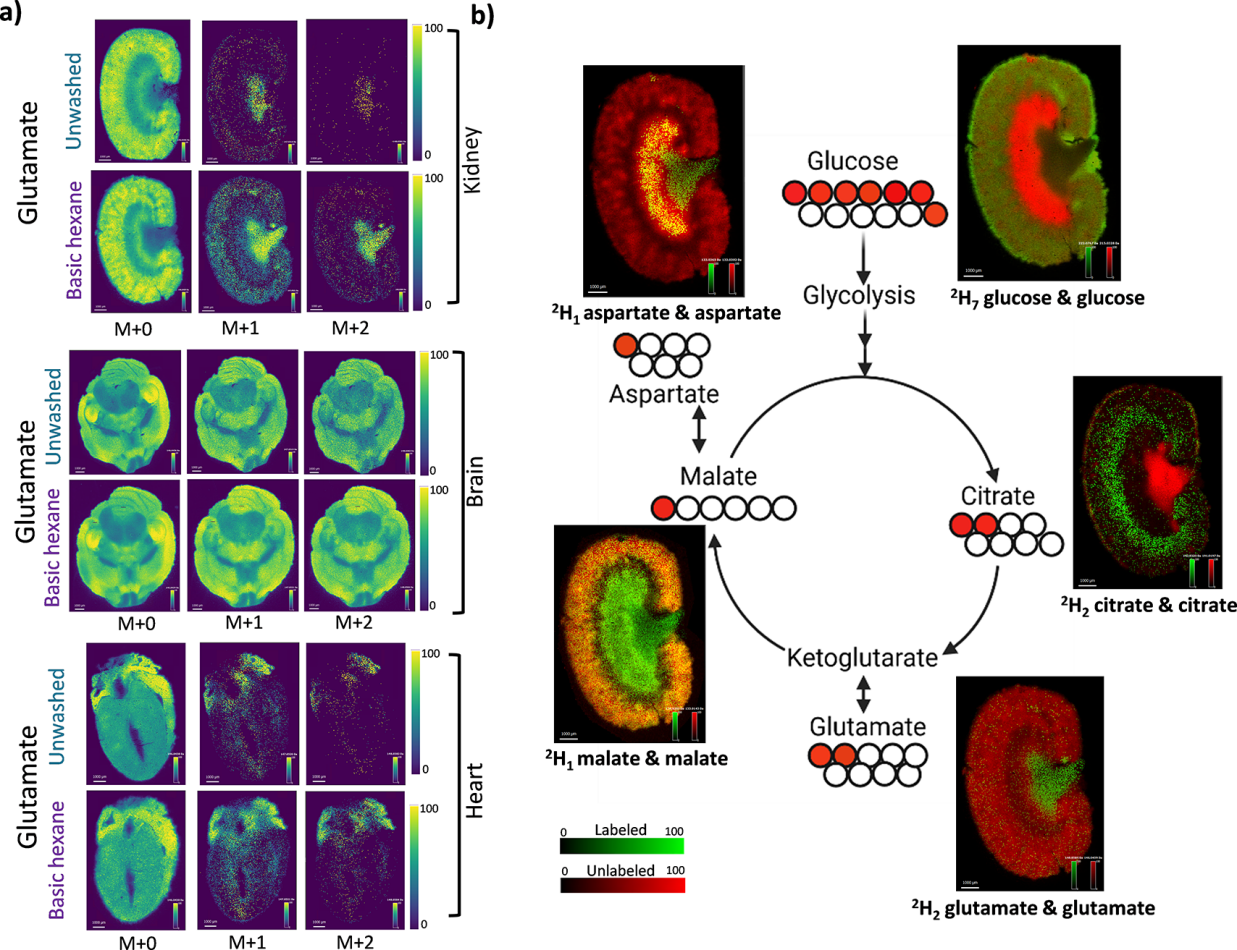
(a) Improved imaging of ^2^H-labeled glutamate and its isotopologue images in the kidney, brain, and heart tissues after basic hexane wash. (b) Pathway mapping of ^2^H-labeled and unlabeled metabolites in the kidney identified after basic hexane wash. The overlay of labeled (green) and unlabeled (red) color shows a region-specific possible distribution of infused and endogenous metabolites. The yellow color represents the presence of both unlabeled and labeled isotopologues of the same metabolite. Scale bar: 1 mm. The isotopologue distribution of ^2^H labels attached to their respective carbon positions is shown with small red circles and is tentatively assigned.

In our study, we encountered some limitations inherent in the basic hexane wash for molecular coverage and delocalization. Although the basic hexane wash is effective in removing lipids from tissues, it may be inadequate for certain nonpolar metabolites such as palmitate, oleate, and other fatty-acid-like molecules. Also, the delocalization effects for these metabolite classes must be carefully evaluated using orthogonal technologies such as LMD (described earlier) and MALDI-immunohistochemistry (MALDI-IHC). When combined with other molecular omics analyses, it can provide complementary insights about related enzyme levels and spatial biochemical processes. Furthermore, combining this method with on-tissue derivatization^36^ ^,^ ^37^ and new MALDI matrices^38^ can improve sensitivity to allow metabolite imaging at even lower pixel sizes of cellular resolution. Although ^2^H-glucose labeling can provide insights into the organ metabolism, the lack of dynamic blood sampling in this study limits the interpretation of the results.

## Conclusions

Polar metabolite imaging in organ tissues is currently limited due to its low MALDI-MS sensitivity and ion suppression from abundant molecules. We have developed a basic hexane tissue pretreatment in combination with the NEDC matrix that remarkably improved the sensitivity of polar metabolites in five organ tissues covering key metabolic pathways. We have also demonstrated the spatial validation approach as a proof of concept, addressing the challenge of potential delocalization caused by washing and providing the LMD-LCMS/MS workflow to decipher metabolic heterogeneity within tissues. Our method is simple, is reproducible, and outperformed existing sample treatment methods in all tested tissue types. Importantly, for the first time, we report the improved imaging of ^2^H-labeled metabolites with spectral specificity. We validated the method for reproducibility of metabolite signals and their identifications. This comprehensive and validated approach of basic hexane wash combined with other approaches can achieve unprecedented sensitivity and can enable the imaging of metabolites at cellular resolution.

## Supplementary Material







## Data Availability

MALDI-MSI raw data are available at the METASPACE repository: https://metaspace2020.eu/project/swapna-2024. LC-MS raw data are available at the MassIVE repository: MassIVE MSV000096852; https://massive.ucsd.edu/ProteoSAFe/dataset.jsp?task=cc12305719e6400c8a7e36ef97fccbbc.

## Notes

Supplementary figure information including solvent postwash analysis, solvent mixtures studied, optimized basic additive concentrations and the volume of basic hexane required, ionization efficiency of selective metabolites at varying pH ranges, H&E staining images, comparative heatmap of metabolite signal intensities from all five organ tissues studied, LMD LC-MS/MS method optimization on the liver, comparison of MALDI-MSI and LMD-LC-MS/MS analysis in three distinct tissue regions of the kidney, and MS ion images of labeled metabolites annotated in all studied tissues (PDF)
Supplementary method information including materials, animal experimentation, tissue preparation, matrix application, data acquisition and analysis using MALDI-MS imaging, hematoxylin and eosin (H&E) staining, tissue sampling by laser capture microdissection, sample preparation, LC-MS/MS-based method, data analysis for LMD-LC-MS/MS metabolomics, BCA protein assay, and comparison between MALDI-MSI and LMD-LC-MS/MS analysis (PDF)
Summary of metabolite coverage from various wash conditions tested from each tissue type studied, pathway mapping, BCA protein estimation of liver tissue post wash and LMD kidney tissue sections, list of unlabeled and labeled metabolites detected among tissue types upon different solvent pretreatment, comparative metabolite intensity values from LipostarMSI and METASPACE, reproducibility of basic hexane wash protocol in kidney tissues, on-tissue MALDI-MS/MS, optimization of the liver tissue for LMD-LCMS workflow pre- and postbasic hexane, and region-specific metabolite abundances from MALDI and LMD-LCMS of the kidney washed with basic hexane (XLSX)
