# Supplementary material for: Improved MALDI-MS Imaging of Polar and 2H‑Labeled Metabolites in Mouse Organ Tissues
Source: Anal Chem. 2025 May 13;97(20):10720–8. doi: 10.1021/acs.analchem.5c00620 (PMC12120821; doi:10.1021/acs.analchem.5c00620)
Supplement: Supplementary file 1 [file ac5c00620_si_001.pdf]

Supporting Information

Supplementary Figures

Improved MALDI-MS Imaging of Polar and ^{2}H-Labeled Metabolites in Mouse Organ Tissues

Siva Swapna Kasarla[1], Antonia Fecke[1], Karl William Smith[1], Vera Flocke[2,3], Ulrich Flögel[2,3], Prasad Phapale[1,2]*

1. Leibniz-Institut für Analytische Wissenschaften—ISAS—e.V., Otto-Hahn-Str. 6b, Dortmund 44227, Germany

2. Experimental Cardiovascular Imaging, Institute for Molecular Cardiology, Heinrich Heine University Düsseldorf, Düsseldorf 40225, Germany

3. Cardiovascular Research Institute Düsseldorf (CARID), Düsseldorf 40225, Germany

4. Department of Environmental Science, Aarhus University, Frederiksborgvej 399, Roskilde, 4000, Denmark

*Corresponding author: prasad.phapale@envs.au.dk

Table of contents:

Figure S1: MALDI-MS analysis of solvents analysis of post hexane and chloroform wash-----
---(Page S3)
Figure S2: Tentative annotation of lipids based on exact mass from Figure S1--------(Page S4)
Figure S3: Combination of solvent mixtures studied in liver tissue---------------------(Page S5)
Figure S4: Optimization of basic additives in heart tissue--------------------------------(Page S6)
Figure S5: Effect of basic hexane wash volumes on lipid removal-----------------------(Page S7)
Figure S6: Ionization efficiency of metabolites with and without additives in MALDI-MSI----
---(Page S8)
Figure S7: Effect of additives in ionization of metabolites in MALDI-MSI------------(Page S9)
Figure S8: Improvement in pyruvate signal across five tissues after basic hexane wash--------
--(Page S10)
Figure S9: Effect of solvent wash on tissue integrity----------------------------------(Page S11)
Figure S10: Heatmap of the signal intensities of all annotated metabolites among different solvent wash conditions in the kidney---(Page S12)
Figure S11: Heatmap of the signal intensities of all annotated metabolites among different solvent wash conditions in the heart--(Page S13)
Figure S12: Heatmap of the signal intensities of all annotated metabolites among different solvent wash conditions in the liver---(Page S14)
Figure S13: Heatmap of the signal intensities of all annotated metabolites among different solvent wash conditions in the brain--(Page S15)
Figure S14: Heatmap of the signal intensities of all annotated metabolites among different solvent wash conditions in BAT--(Page S16)
Figure S15: Comparative heatmap of metabolite intensities obtained from two different MS imaging analysis software---(Page S17)
Figure S16: Effect of basic hexane wash in positive- and negative- ion mode-------(Page S18)
Figure S17: MALDI-MS spectra showing well resolved labeled deuterium peaks from nature ^{13}C isotopic peaks--(Page S19)
Figure S18: Effect of basic hexane wash on the liver tissue excised using LMD followed by LC-MS/MS---(Page S20)
Figure S19: PCA score plots from MALDI-ROI and LMD-ROI------------------------(Page S21)
Figure S20: Comparative box plots of commonly identified metabolites from region specific MALDI-MSI and LMD-LC-MS/MS analysis--(Page S22-27)
Figure S21: MS ion images of ^{2}H labeled metabolites annotated in mouse kidney tissue--------
--(Page S28)
Figure S22. MS ion images of ^{2}H labeled metabolites annotated in mouse brain tissue—-------
--(Page S29)
Figure S23: MS ion images of ^{2}H labeled metabolites annotated in mouse heart and liver tissues---(Page S30)
Figure S24: MS ion images of ^{2}H labeled metabolites annotated in mouse BAT tissue---------
--(Page S31)

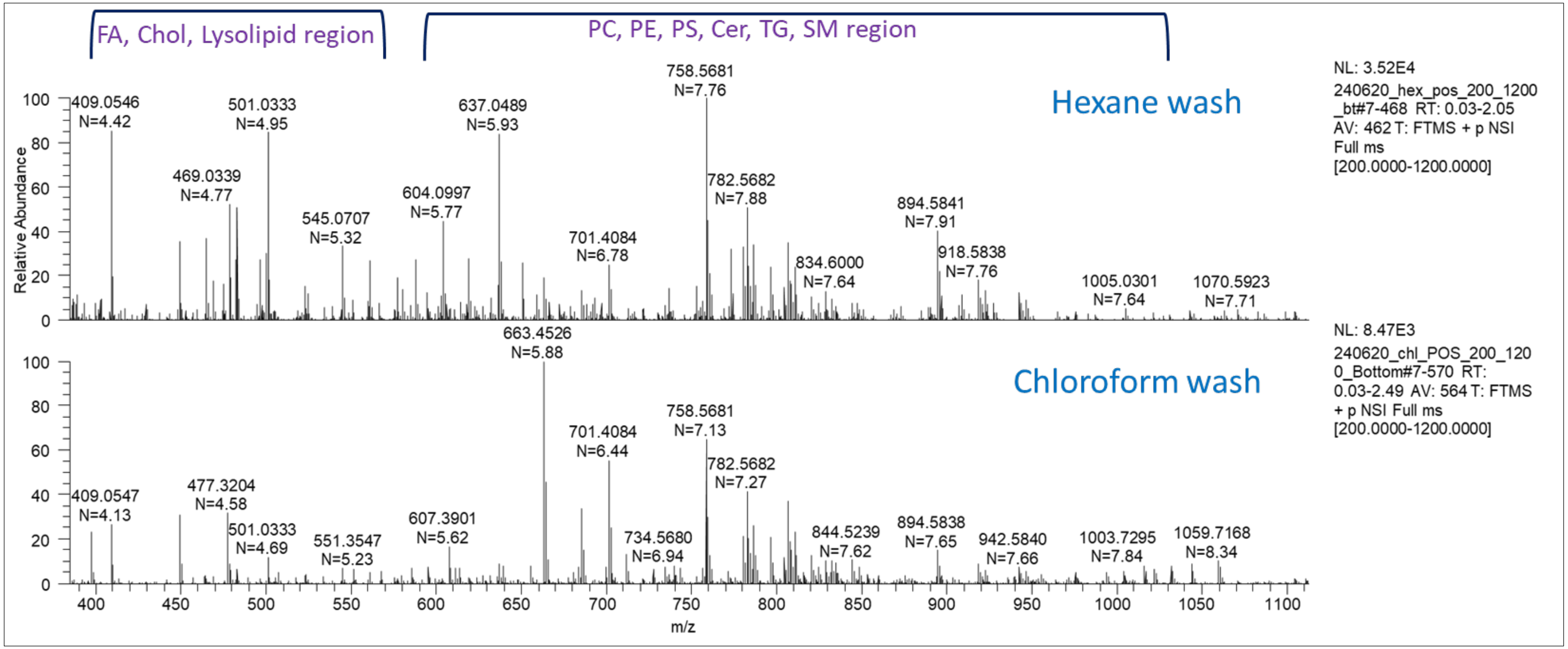

Figure S1: MALDI MS analysis of solvents (chloroforms and hexane) collected post liver tissue wash for: Zoomed mass spectral region of m/z 400-1200 shows higher abundance of lipids. FA: Fatty acids; Chol: Cholesterol; PC: Phosphatidylcholines; PE: Phosphatidylethanolamine; PS: Phosphatidylserine; Cer: Ceramide; TG: Triglycerides; SM: Sphingomyelin

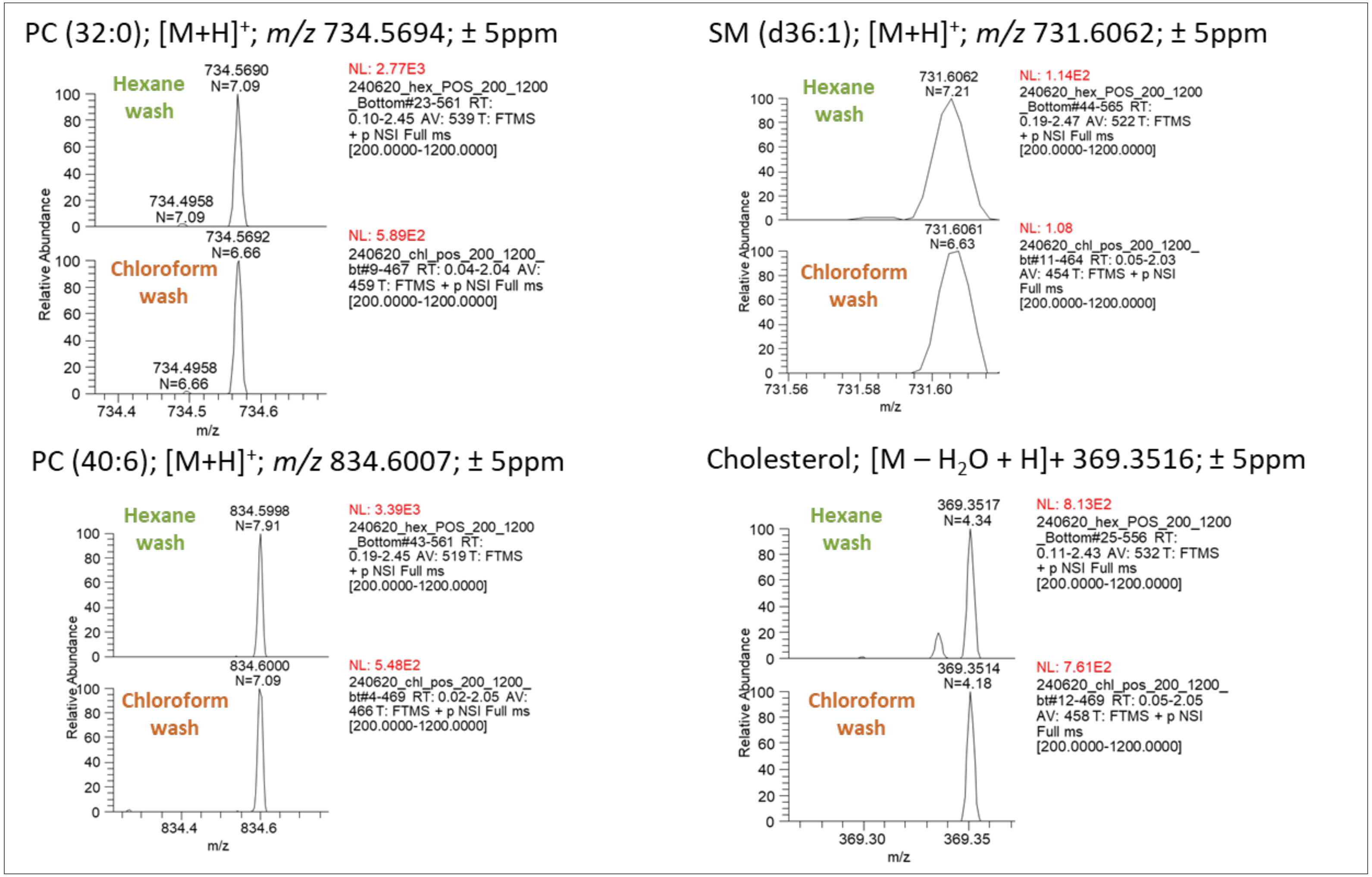

Figure S2: Tentative annotation of lipids based on exact mass from Figure S1, MALDI-MS spectra of annotated lipids and cholesterol. Hexane wash showed higher spectral abundances in the phospholipid ion region (*m/z* 700-900) compared to chloroform (Fig.S1), indicating better lipid removal with hexane for liver tissues.

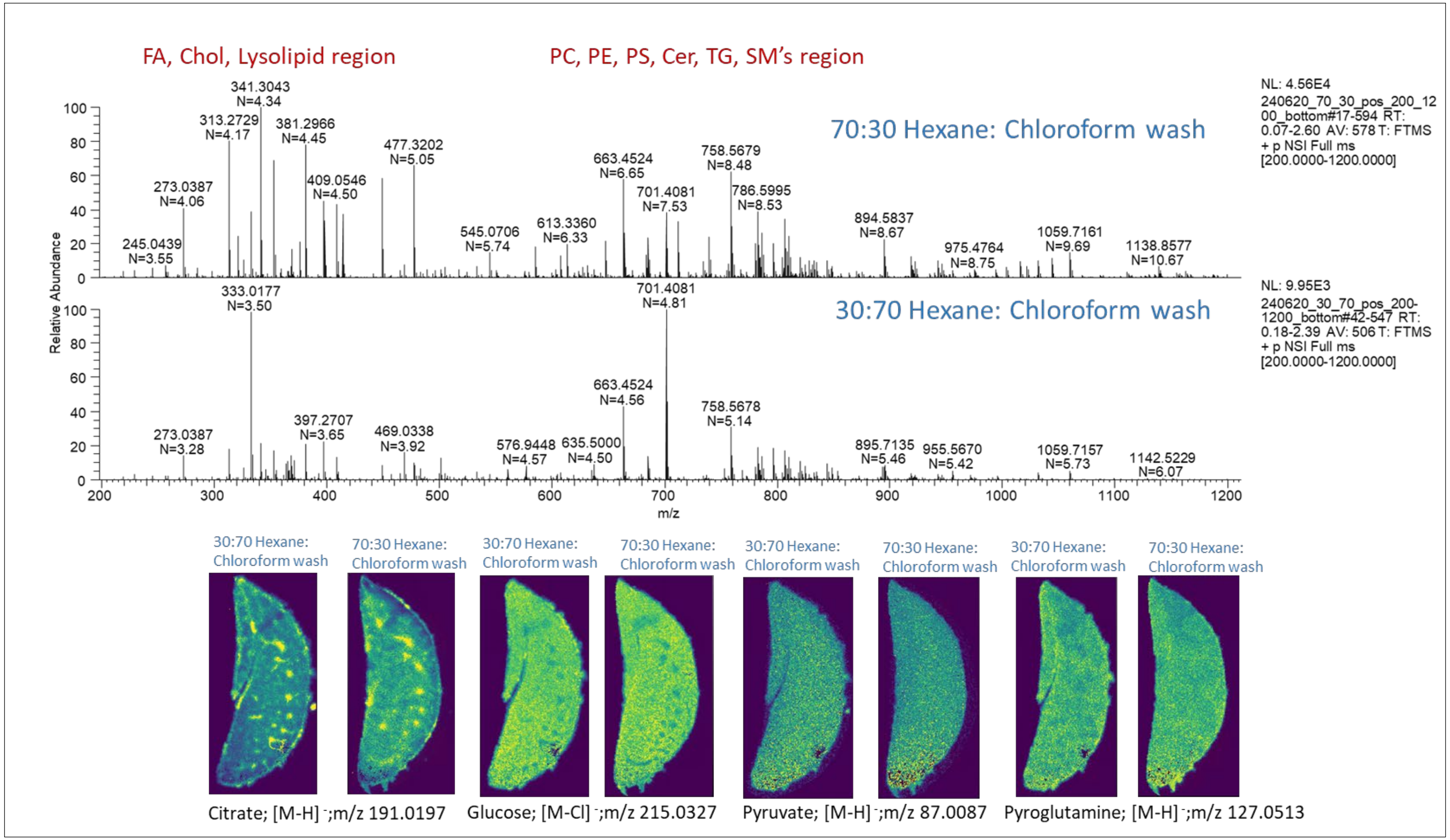

Figure S3: Combination of solvent mixtures (hexane: chloroform) studied in liver tissue: Average mass spectra from the solvent collected post tissue wash with the different ratios of hexane and chloroform mixture and their respective MS ion images for selected metabolites. The data confirming the advantage of using hexane to remove lipids over chloroform. FA: Fatty acids; Chol: Cholesterol; PC: Phosphatidylcholine; PE: Phosphatidylethanolamine; PS: Phosphatidylserine; Cer: Ceramide; SM: Sphingomyelin.

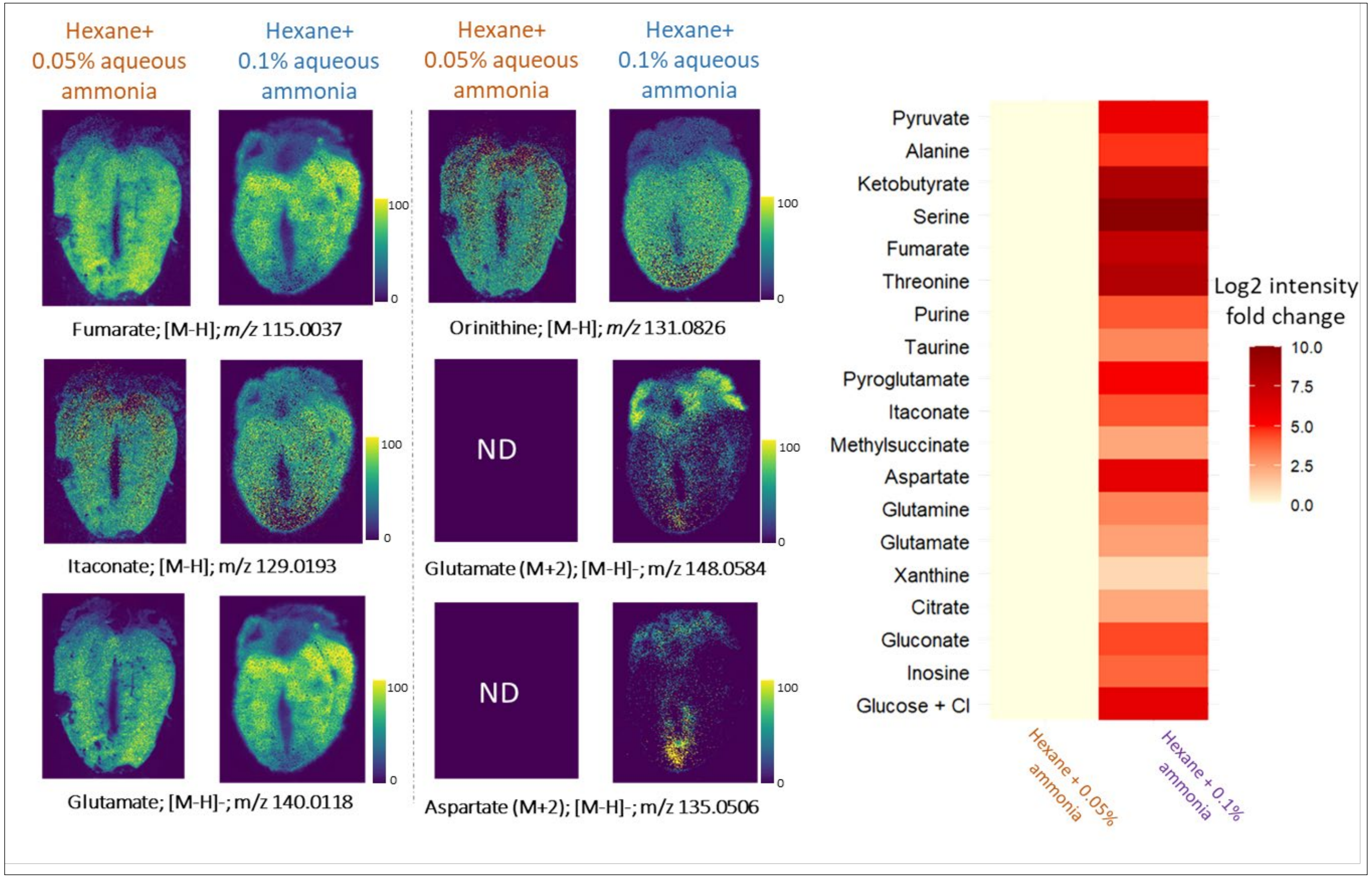

Figure S4: Optimization of basic additives in heart: Metabolite MS ion images with two different conditions of basic additives studied on heart (0.05% and 0.1% aqueous ammonia**).** Heatmap of log base 2 intensity fold changes for selected metabolites showing remarkable effect of basic conditions (0.1 %) for sensitivity improvement.

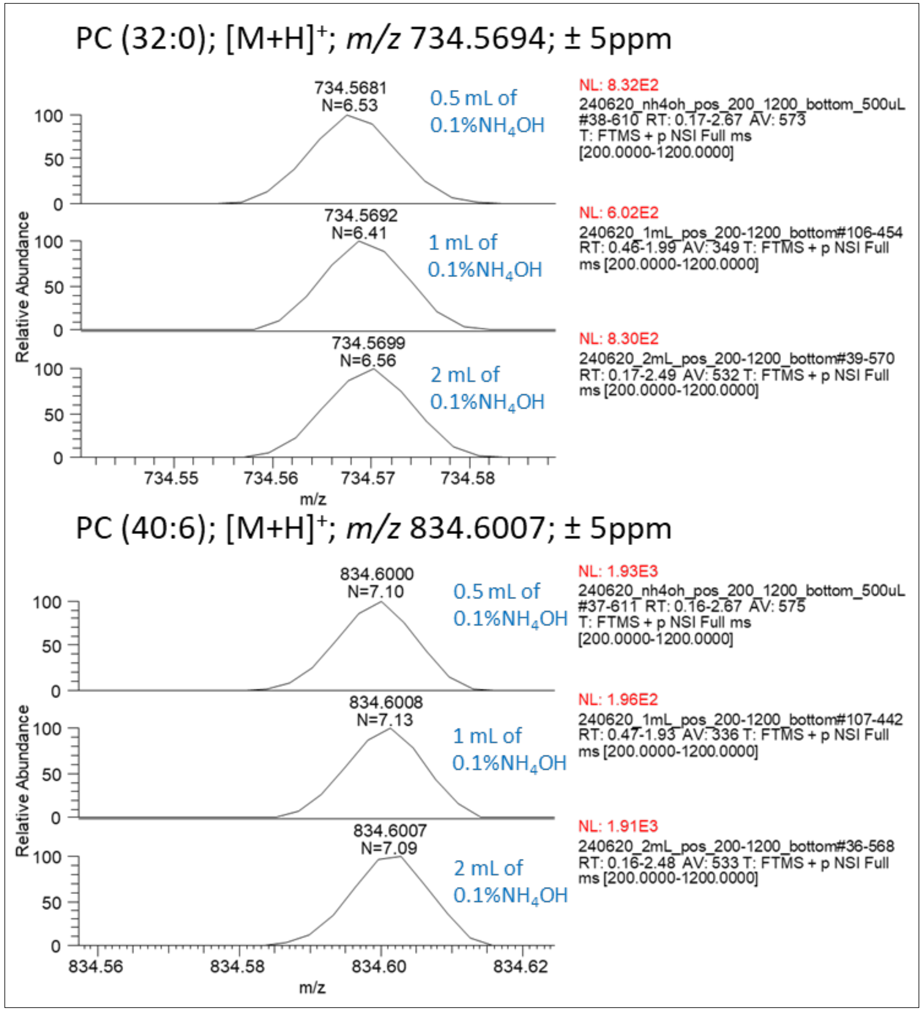

Figure S5: Effect of basic hexane wash volumes on lipid removal: Extracted ion spectra of tentatively annotated lipids from the solvent collected after washing liver tissue with different volumes of basic hexane (0.5 mL, 1mL, 2mL). The spectra show no major differences with increasing solvent volumes.

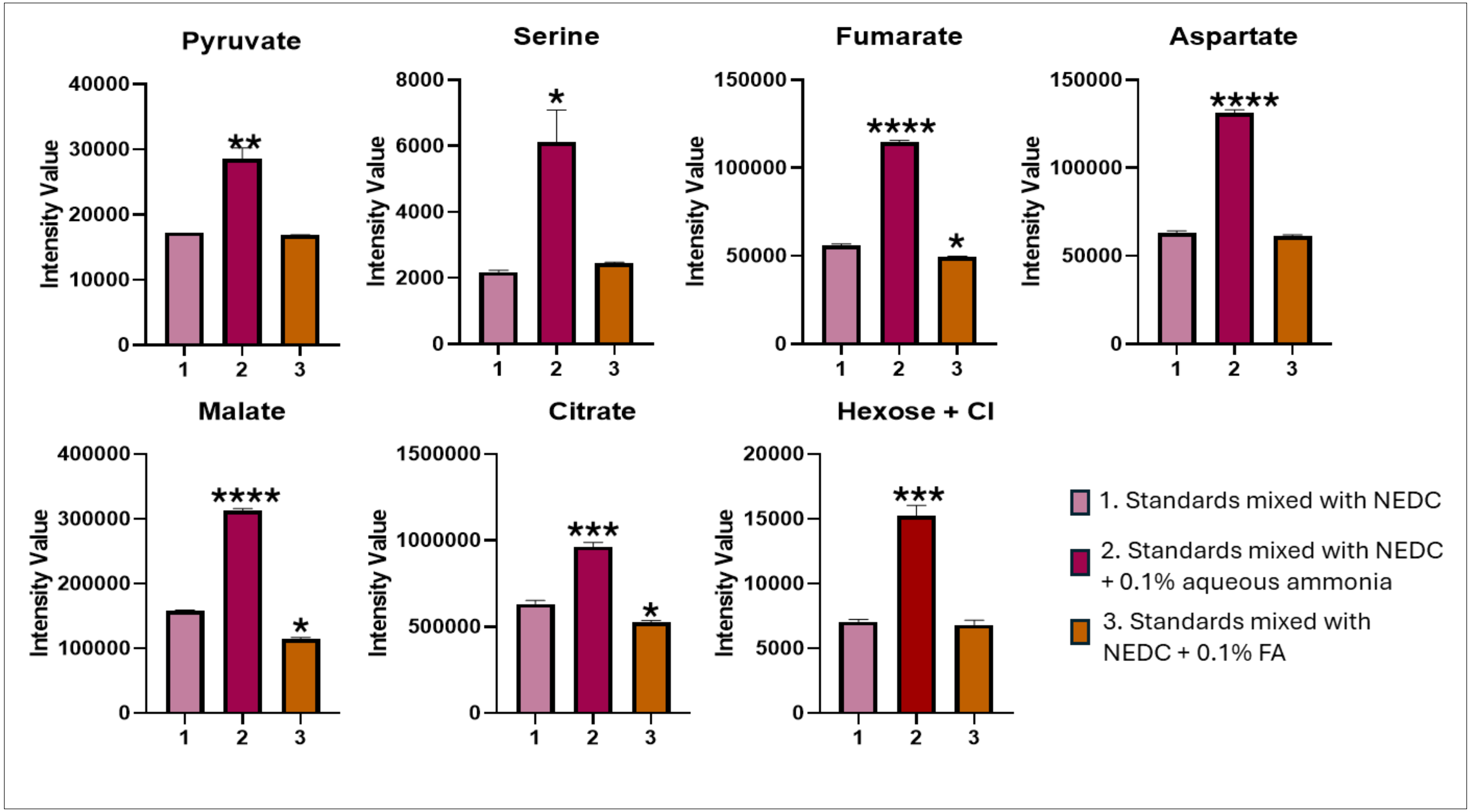

Figure S6: Ionization efficiency of metabolites with and without additives in MALDI-MSI. Selective metabolite standards were mixed in the ratio of 1:3 with NEDC matrix alone, NEDC matrix with basic additive (0.1% aqueous ammonia), NEDC matrix with 0.1% formic acid (FA) and spotted on the MALDI ITO glass slides. The intensity values were extracted from the LipostarMSI software, and one way ANOVA comparison test was performed (n=2). Each group was compared with standards mixed with NEDC; *, **, ***, **** denote p values < 0.05, 0.01, and 0.001, 0.0001 respectively.

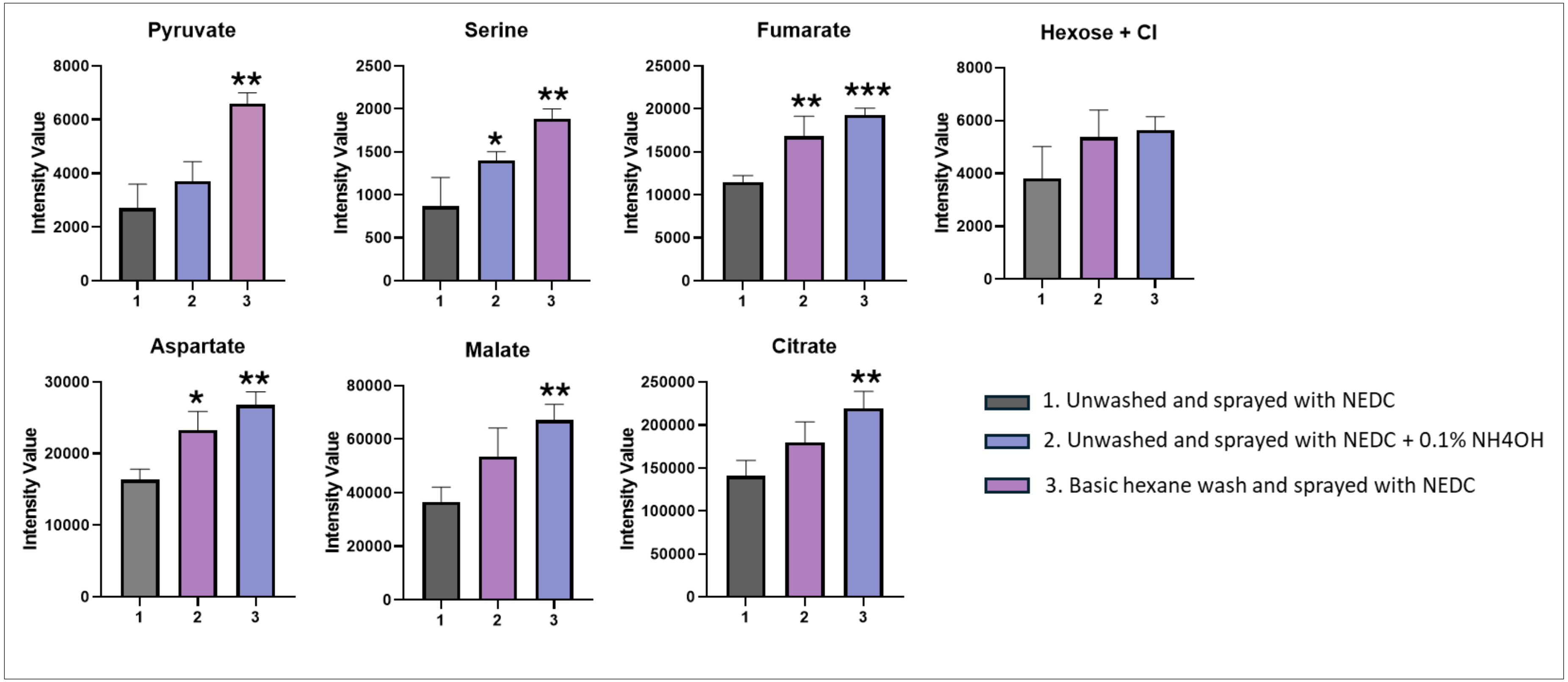

Figure S7: Effect of additives in ionization of metabolites in MALDI-MSI. Selective metabolite standards were spotted on the ITO glass slide. Three different conditions were tested. 1. The standards mixture was spotted and sprayed without any additive or sample pretreatment i.e., “unwashed and sprayed with NEDC”. 2. the spotted standards were sprayed without any washing but with basic additive in the NEDC matrix solution i.e., “unwashed and sprayed with NEDC + 0.1% NH4OH (aqueous ammonia)”. 3. The spotted standards were washed with basic hexane solution and sprayed with regular NEDC solution i.e., “basic hexane wash and sprayed with NEDC”. The intensity values were extracted from the LipostarMSI software, and one way ANOVA comparison test was performed (n=3). Each group was compared with Standards mixed with NEDC; *, **, *** denote p values < 0.05, 0.01, and 0.001 respectively.

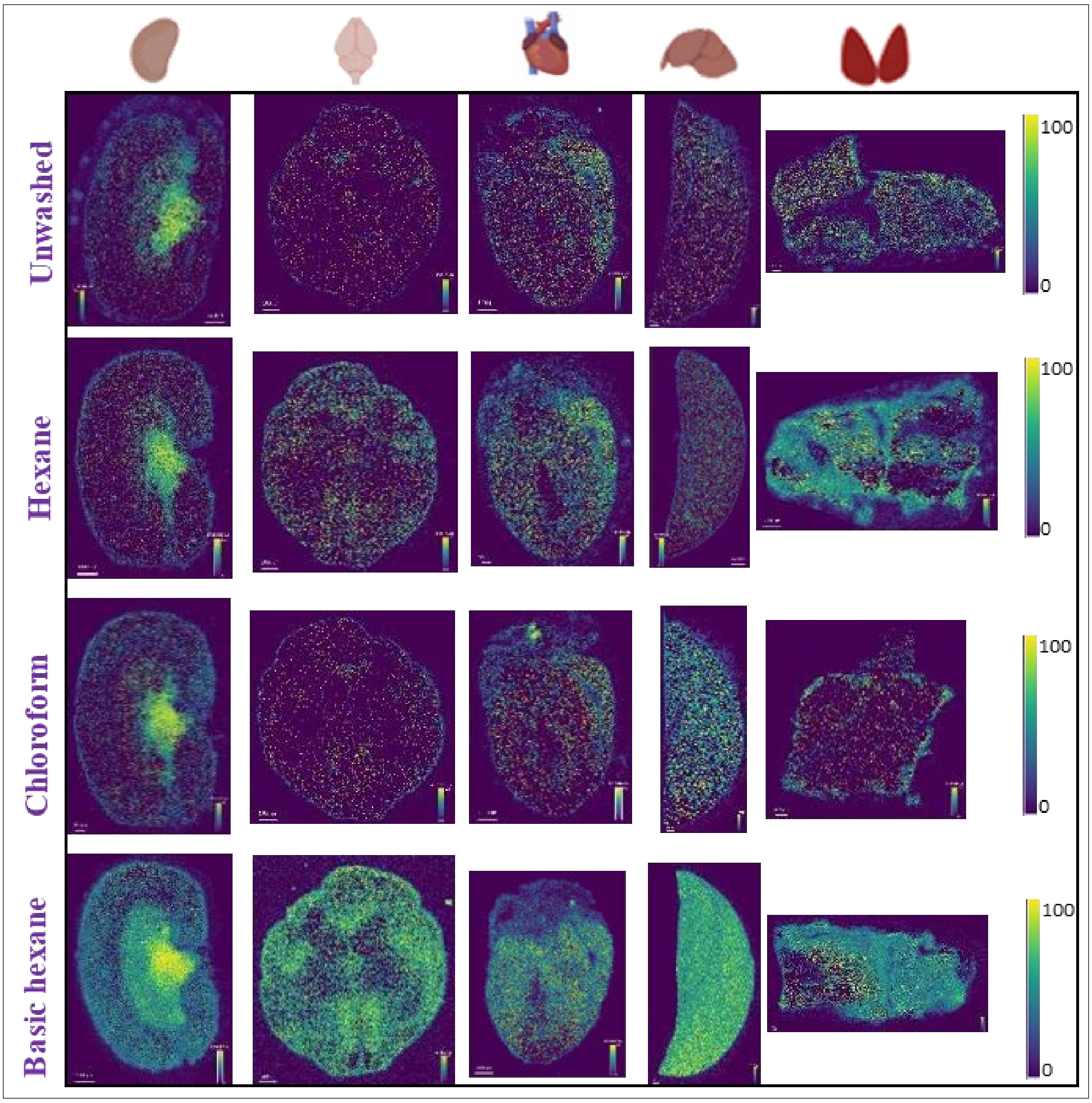

Figure S8: Improvement in pyruvate signal across five tissues after basic hexane wash; Comparative MS ion images of pyruvate showing improved signal detection in all studied tissues.

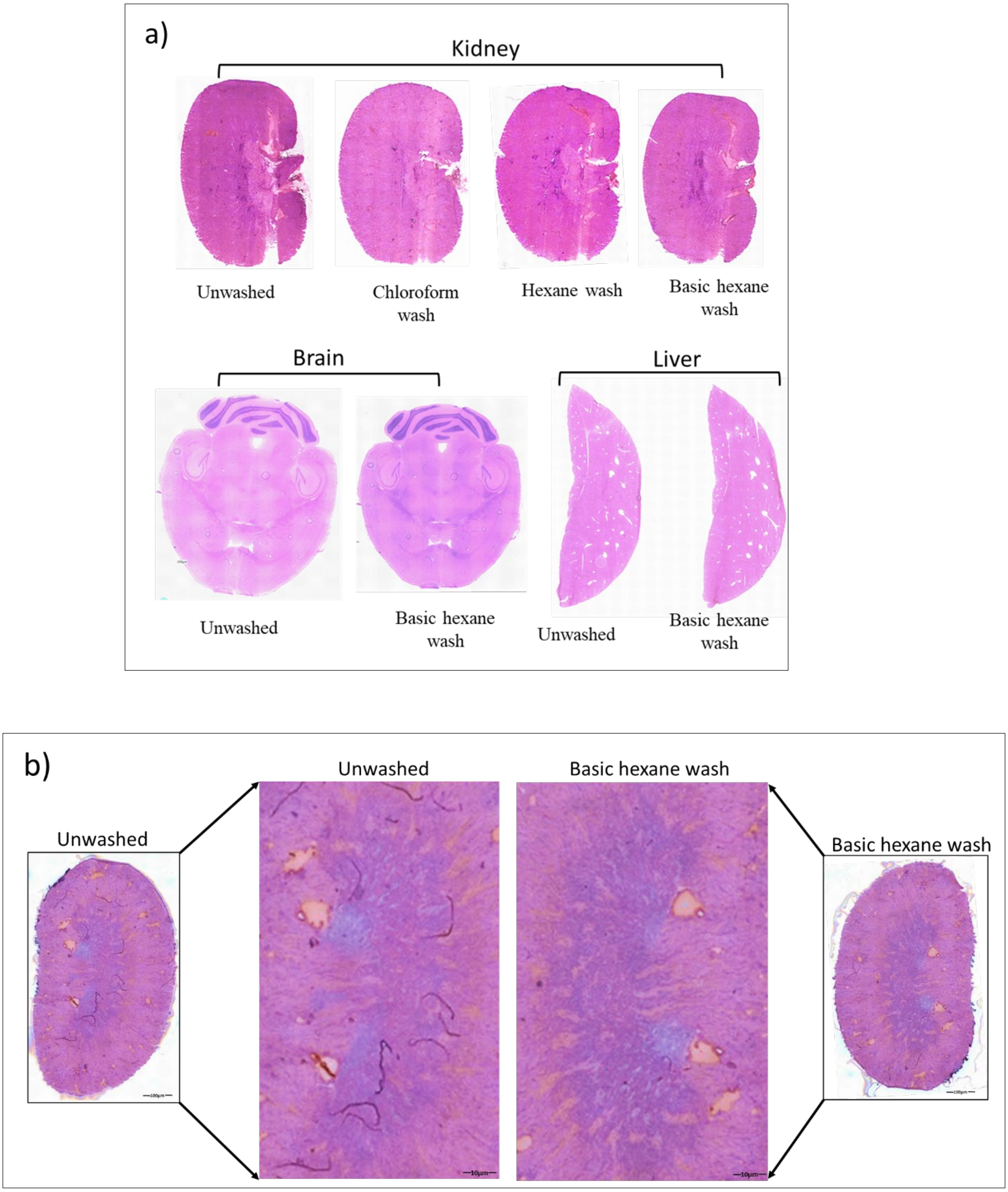

Figure S9: Effect of solvent wash on tissue integrity; a) H&E-stained images of tissues (Kidney, Brain, Liver) before and after wash showed consistent pattern. b) Zoomed H&E-stained kidney image showing no morphological changes.

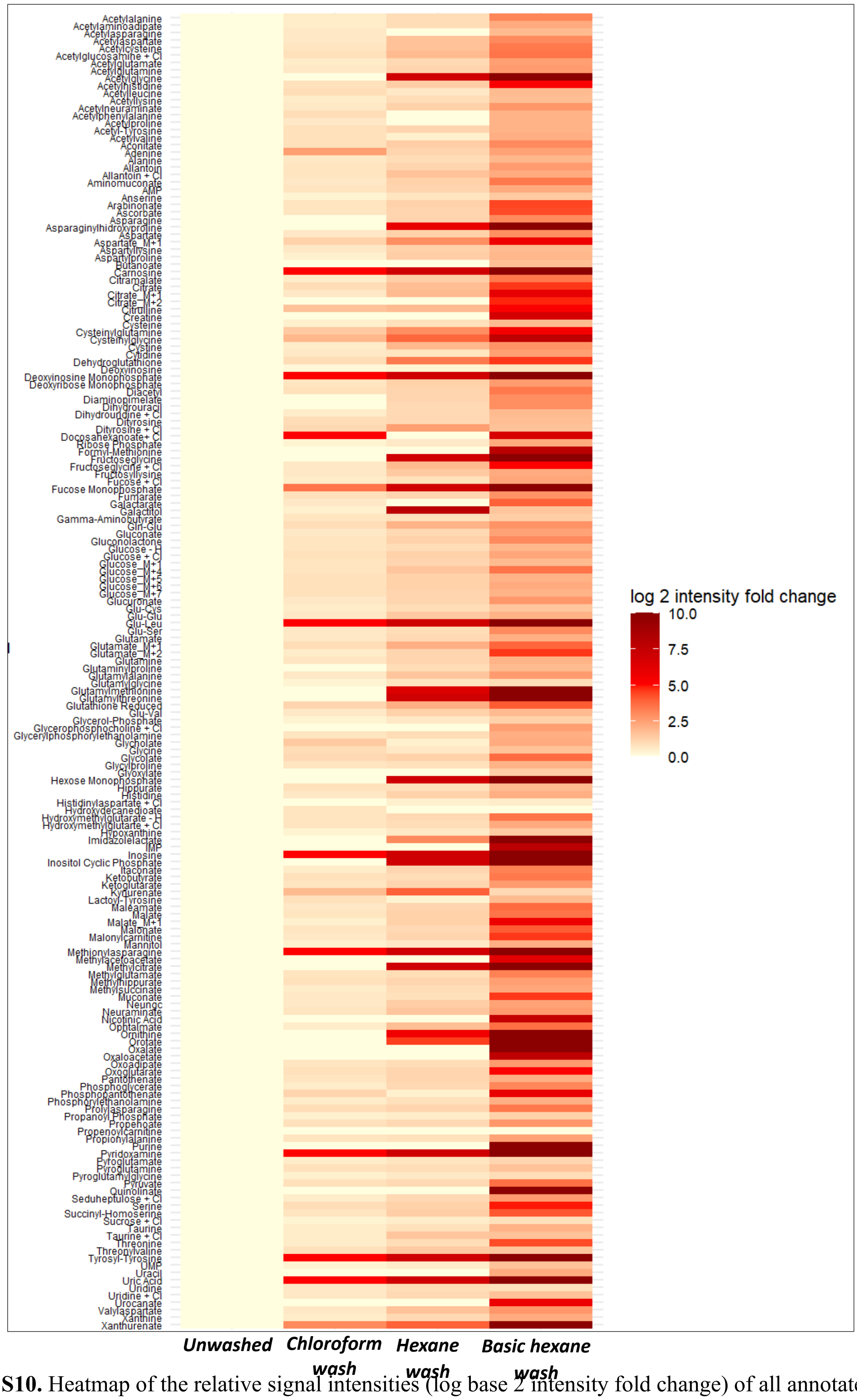

Figure S10. Heatmap of the relative signal intensities (log base 2 intensity fold change) of all annotated metabolites after different wash conditions in kidney tissue.

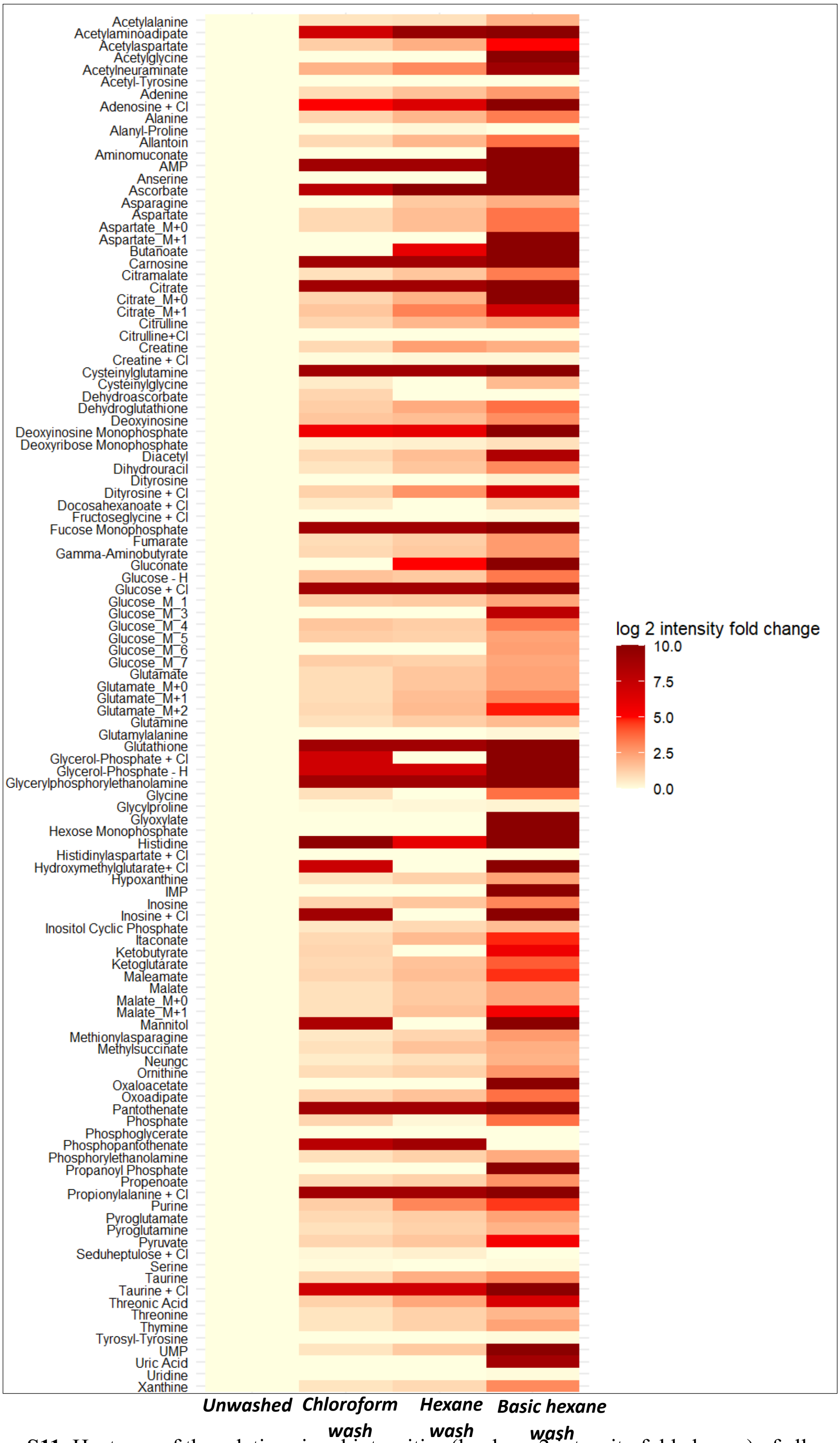

Figure S11. Heatmap of the relative signal intensities (log base 2 intensity fold change) of all annotated metabolites after different wash conditions in heart tissue.

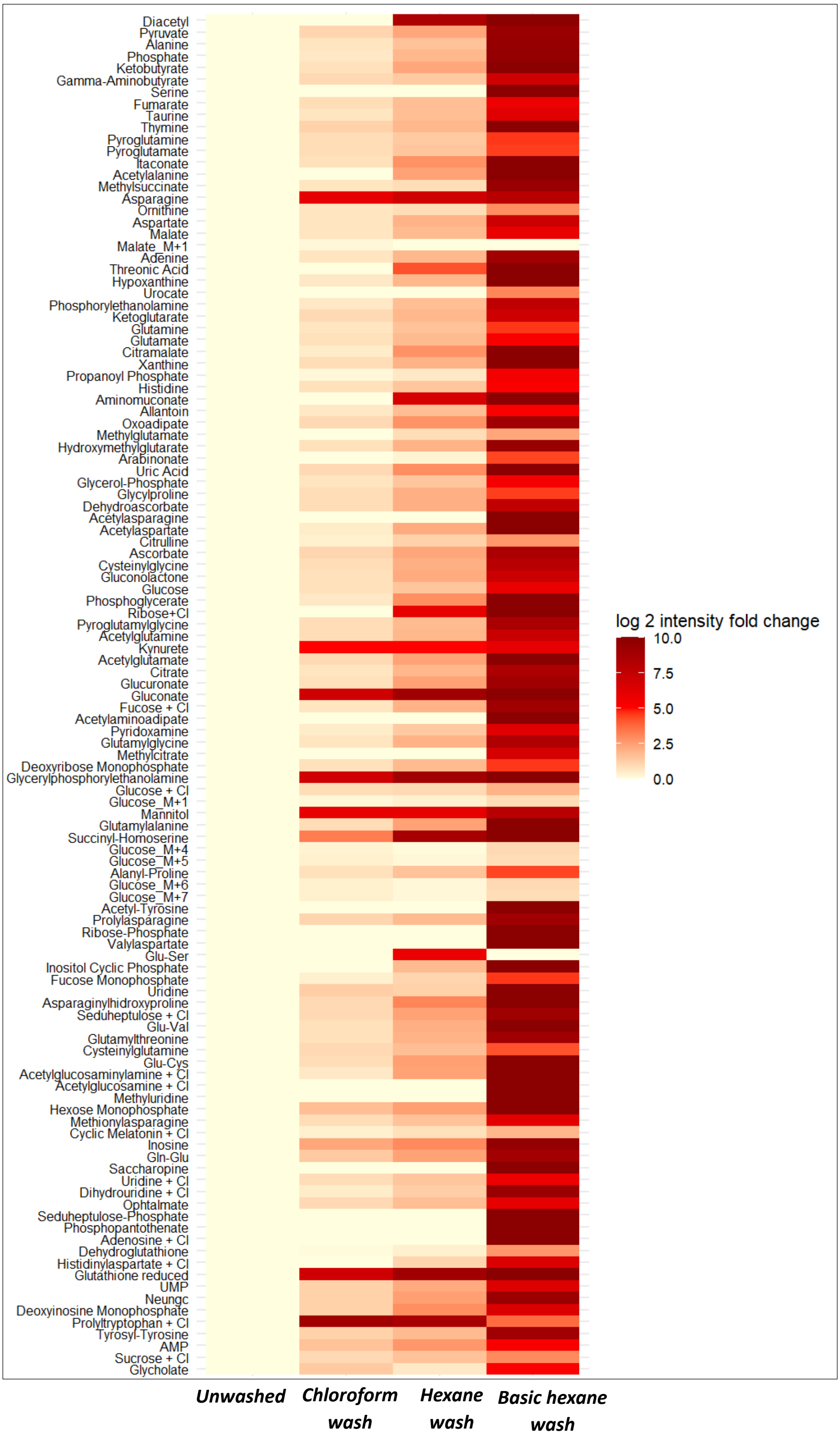

Figure S12. Heatmap of the relative signal intensities (log base 2 intensity fold change) of all annotated metabolites among different wash conditions in liver.

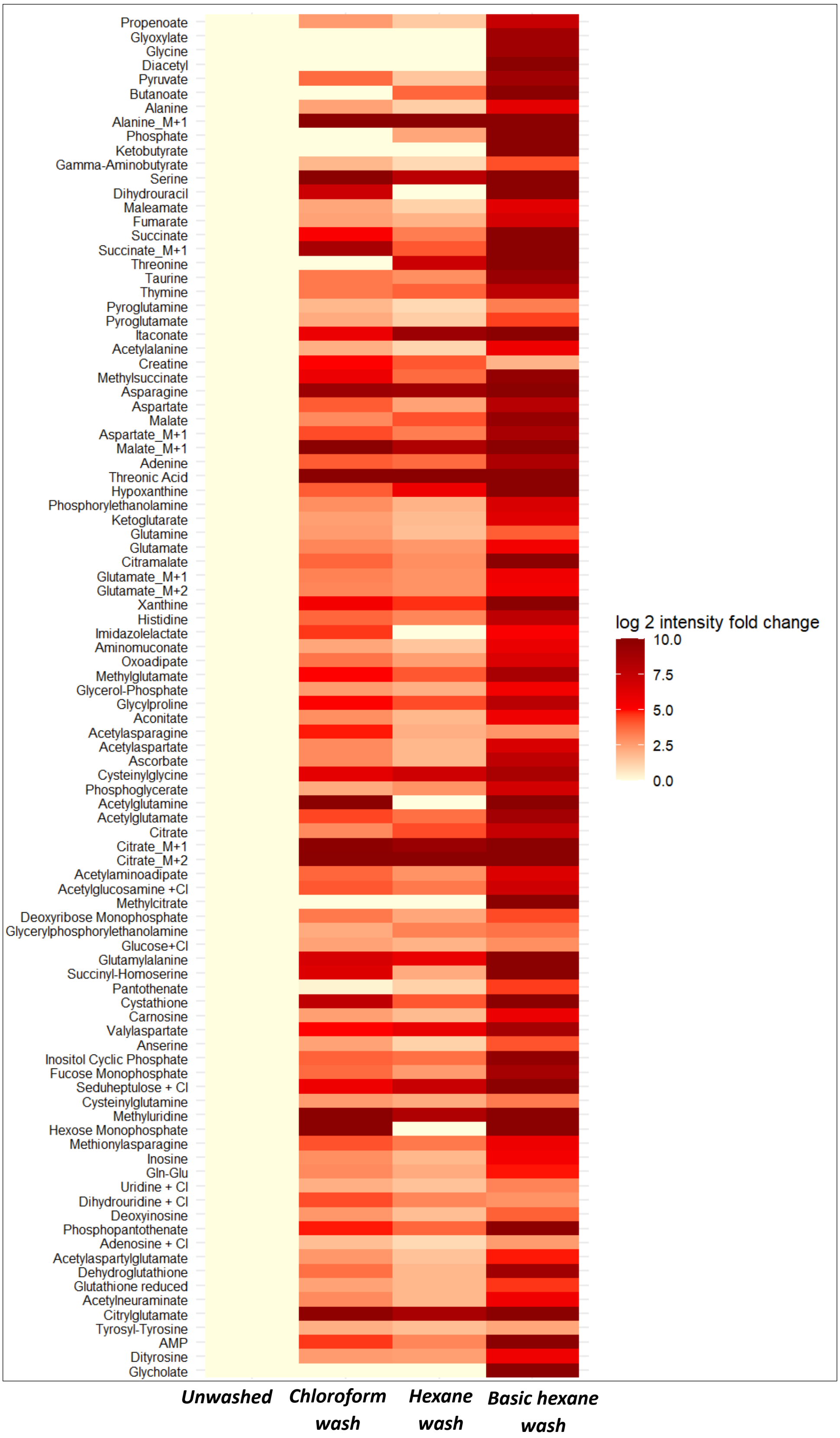

Figure S13. Heatmap of the relative signal intensities (log base 2 intensity fold change) of all annotated metabolites among different wash conditions in brain tissue.

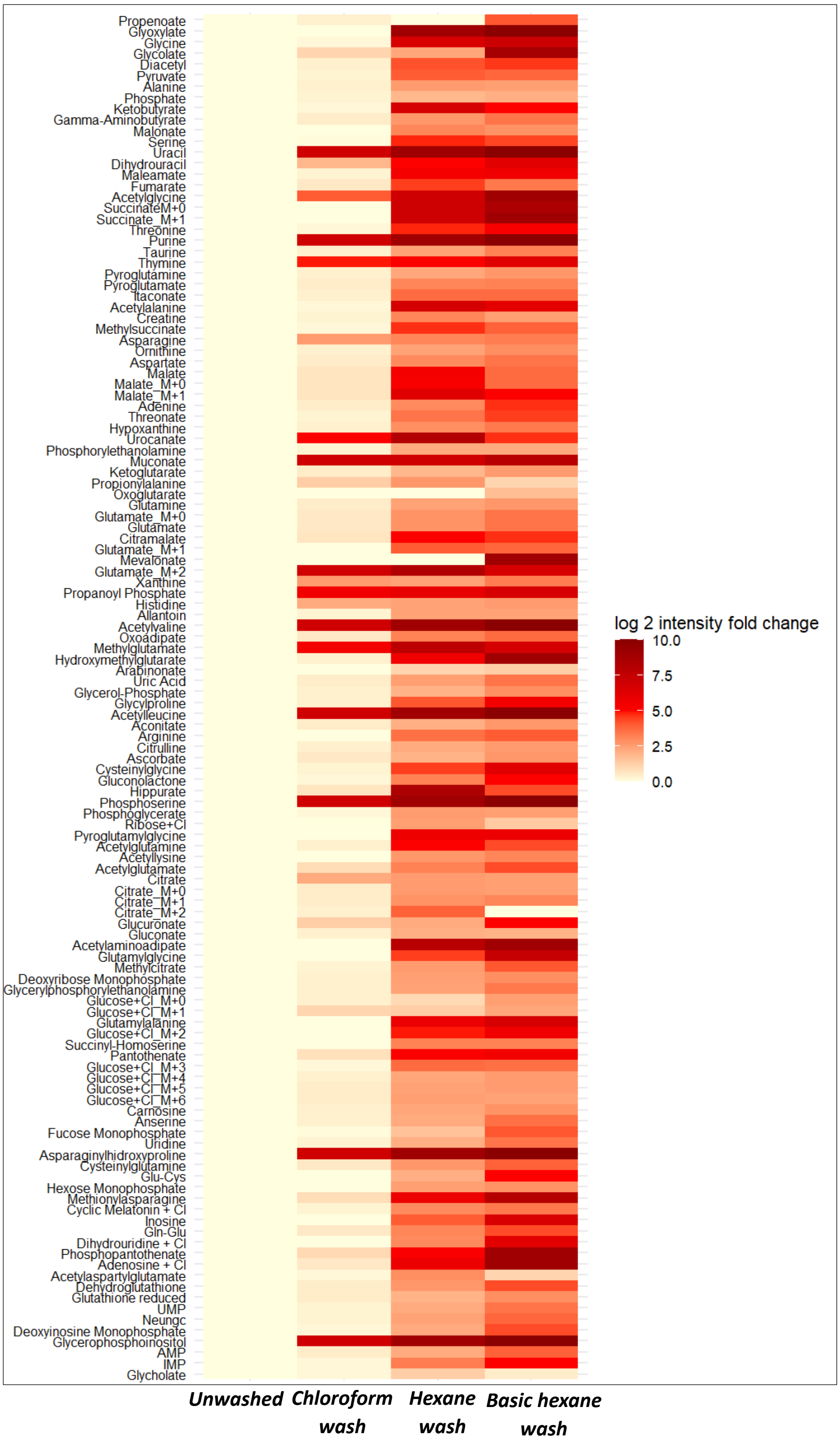

Figure S14. Heatmap of the relative signal intensities (log base 2 intensity fold change) of all annotated metabolites among different wash conditions in BAT tissue.

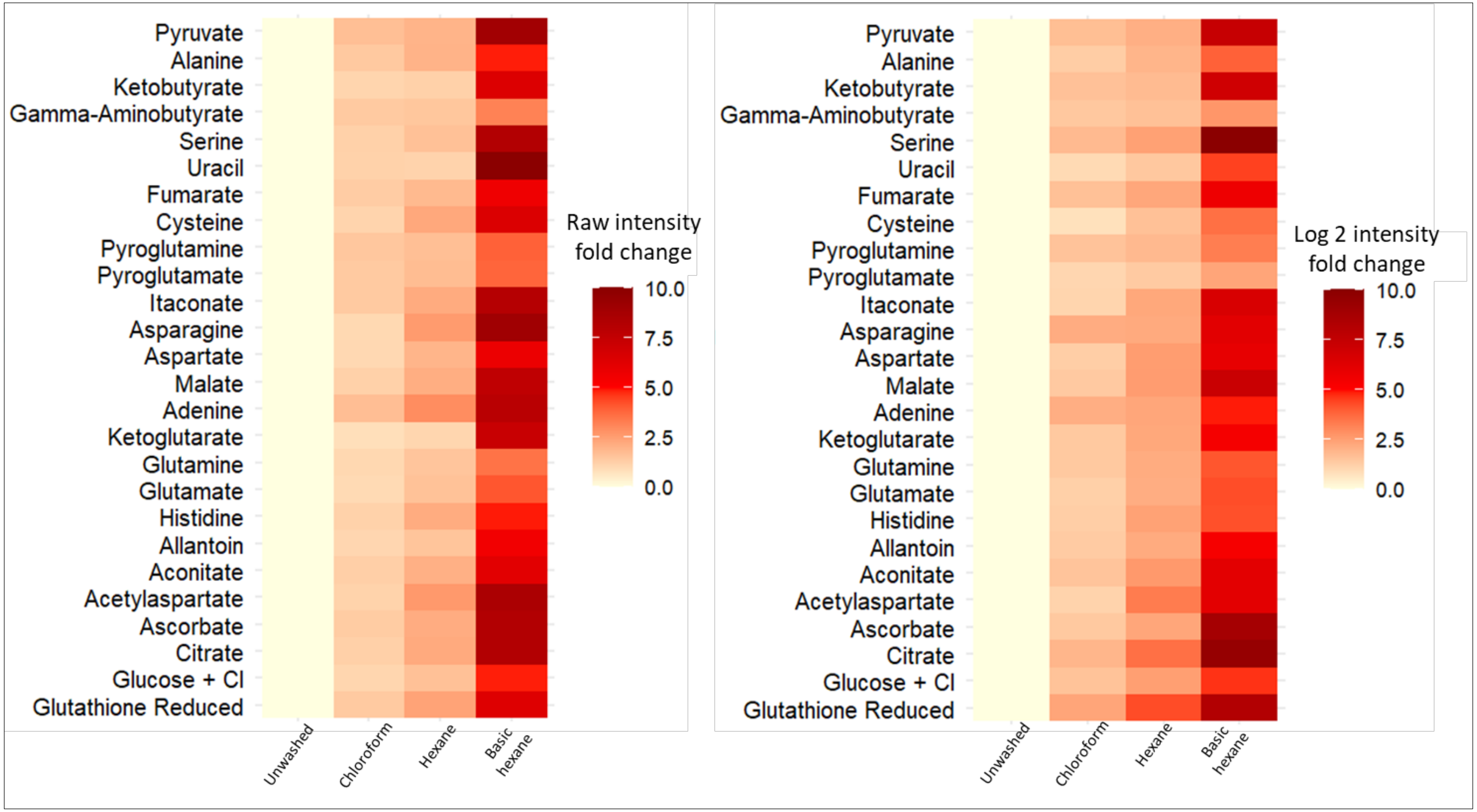

Figure S15: Comparative heatmap of metabolite intensities obtained from two different MS imaging analysis software; a) Raw intensities fold changes from METASPACE b) log 2 intensity fold change from LipostarMSI software.

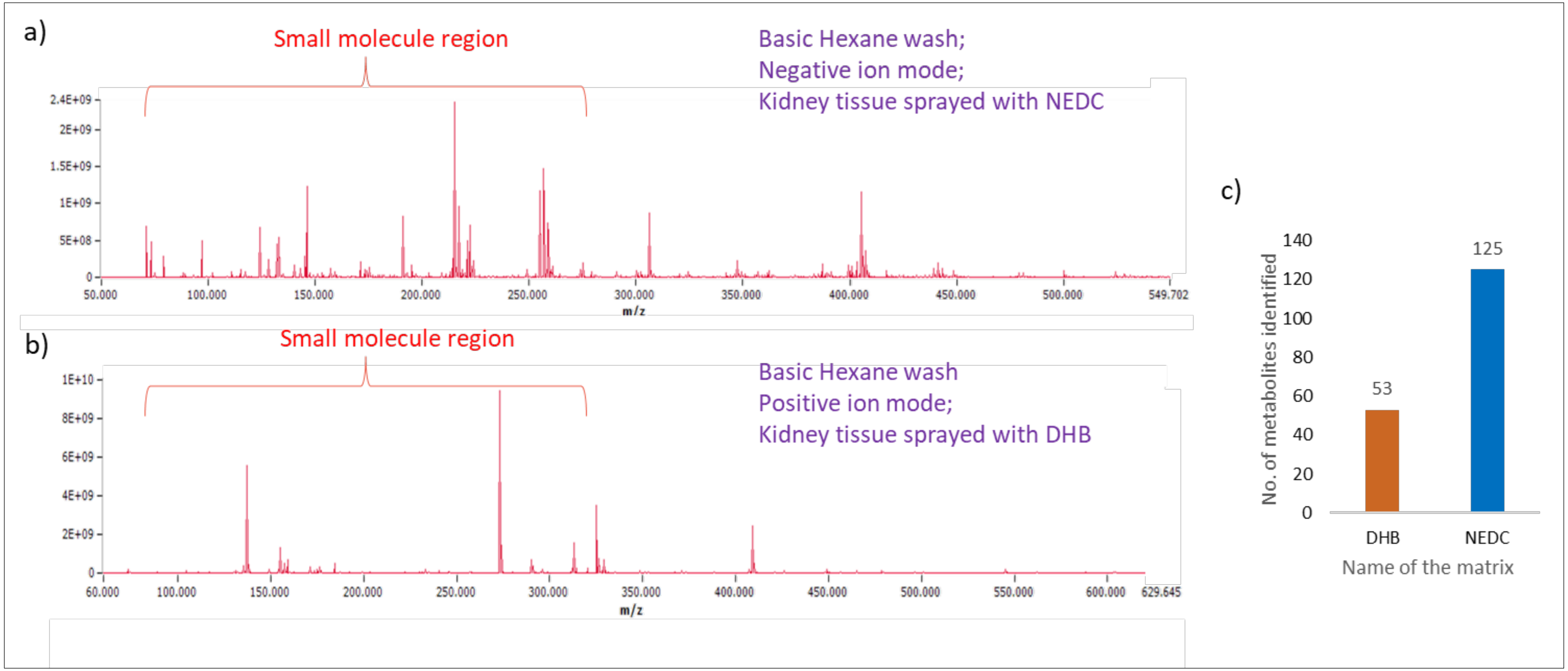

Figure S16: Effect of basic hexane wash in positive and negative ion mode a) Total ion spectra of labeled kidney tissue after basic hexane wash in negative ion mode b) Total ion spectra of labeled kidney tissue after basic hexane wash in positive ion mode. Note: Spectra obtained is from ImageInsight (Spectroglyph LLC) c) comparative bar plot of small molecular polar metabolites annotated in the kidney tissue sprayed with DHB in positive ion mode and NEDC in negative ion mode.

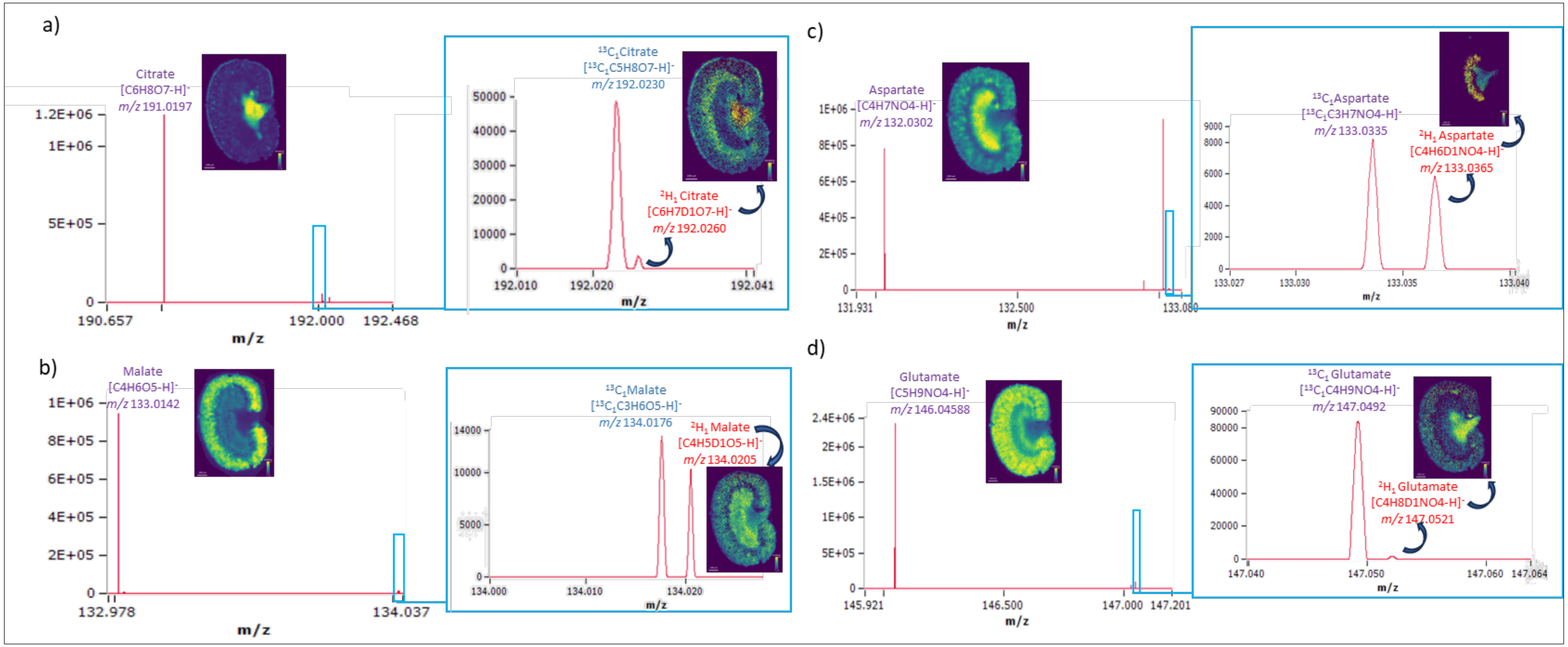

Figure S17. MALDI-MS spectra showing well resolved deuterium labeled peaks from natural ^{13}C isotopic peaks for a) Citrate b) Malate c) Aspartate d) Glutamate at 120,000 orbitrap resolution.

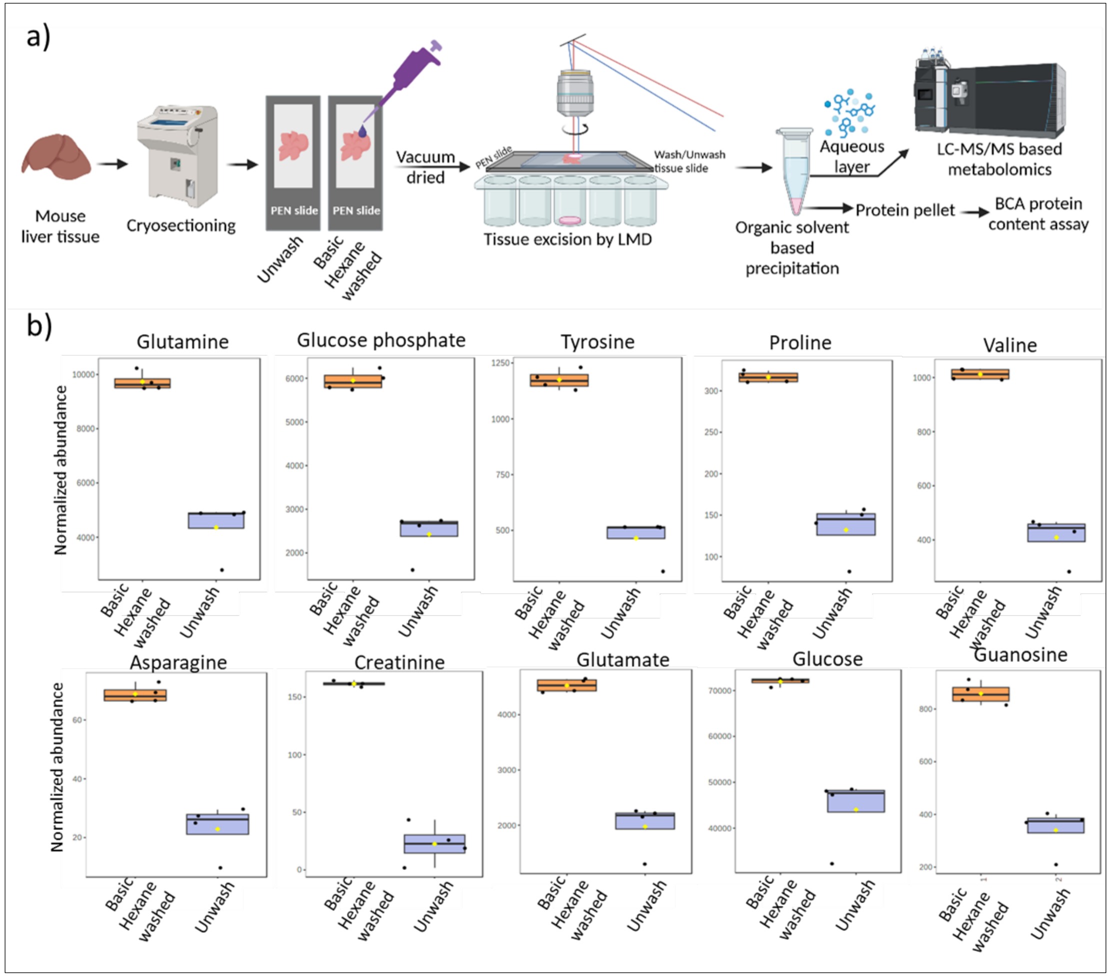

Figure S18: Effect of basic hexane wash on the liver tissue excised using LMD followed by LC-MS/MS a) Workflow depicts mounting the liver tissue on PEN membrane slide followed by basic hexane wash. About 0.8mm^2 area of the tissue was excised for LC-MS/MS based metabolomics b) comparative box plots of metabolites showing significant improvement in the relative intensity in basic hexane washed tissue slide compared with unwashed tissue.

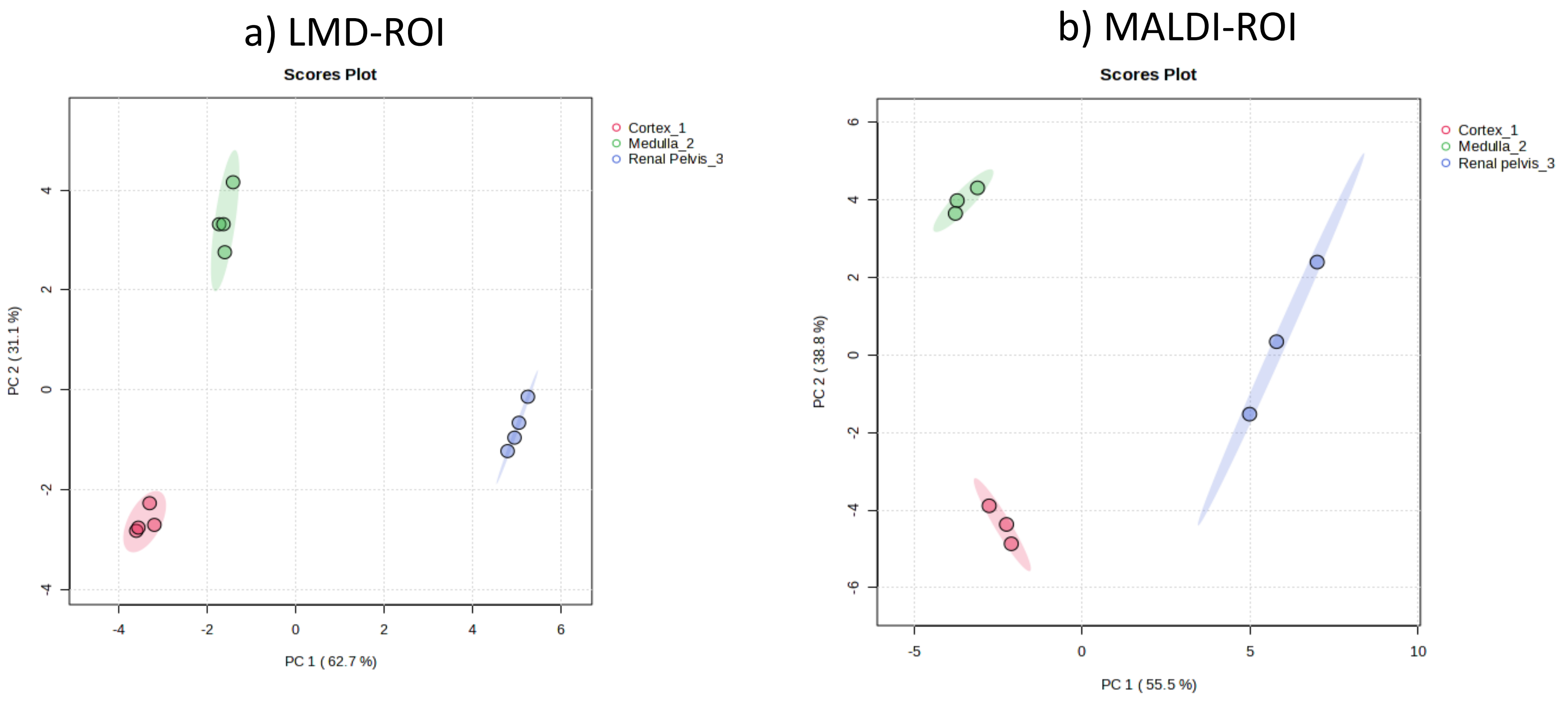

Figure S19. PCA scores plot for a) LMD-ROI b) MALDI-ROI showing consistent grouping and metabolite profile patterns.

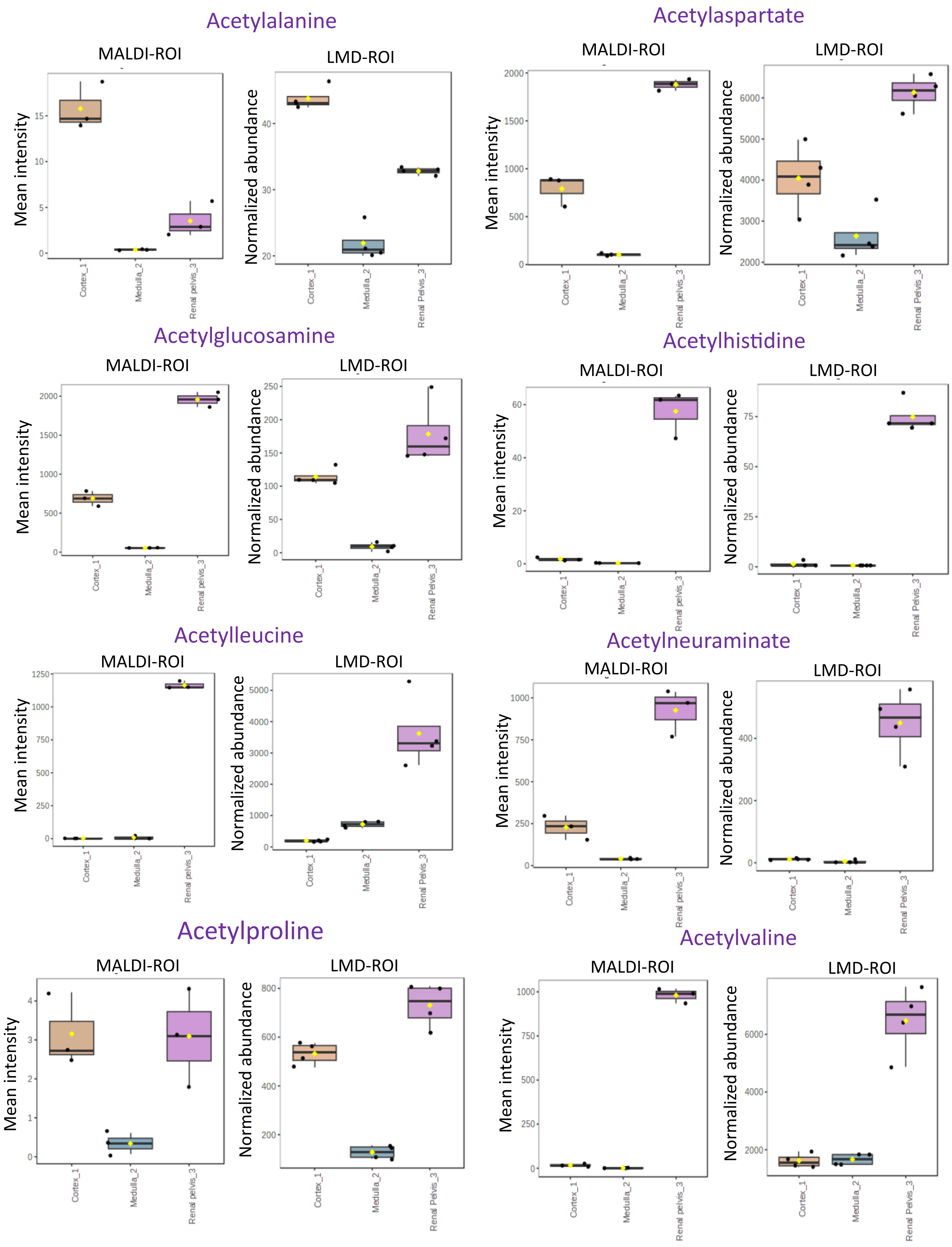
Acetylalanine
MALDI-ROI
Mean intensity
Cortex_1
Medulla_2
Renal pelvis_3
LMD-ROI
Normalized abundance
Cortex_1
Medulla_2
Renal Pelvis_3
Acetylaspartate
MALDI-ROI
Mean intensity
LMD-ROI
Normalized abundance
Acetylglucosamine
MALDI-ROI
Mean intensity
LMD-ROI
Normalized abundance
Acetylhistidine
MALDI-ROI
Mean intensity
LMD-ROI
Normalized abundance
Acetylleucine
MALDI-ROI
Mean intensity
LMD-ROI
Normalized abundance
Acetylneuraminate
MALDI-ROI
Mean intensity
LMD-ROI
Normalized abundance
Acetylproline
MALDI-ROI
Mean intensity
LMD-ROI
Normalized abundance
Acetylvaline
MALDI-ROI
Mean intensity
LMD-ROI
Normalized abundance

Aconitate

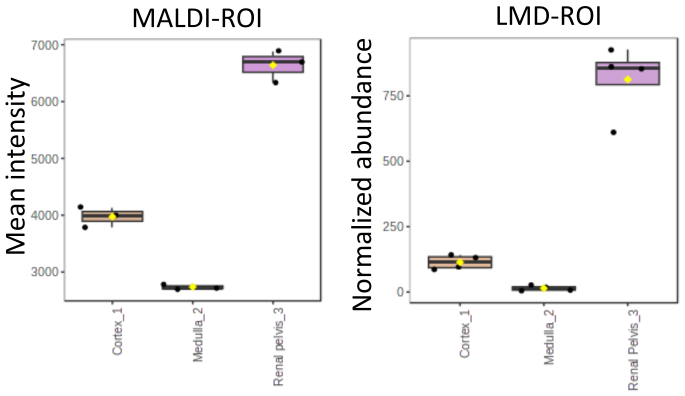

Alanine

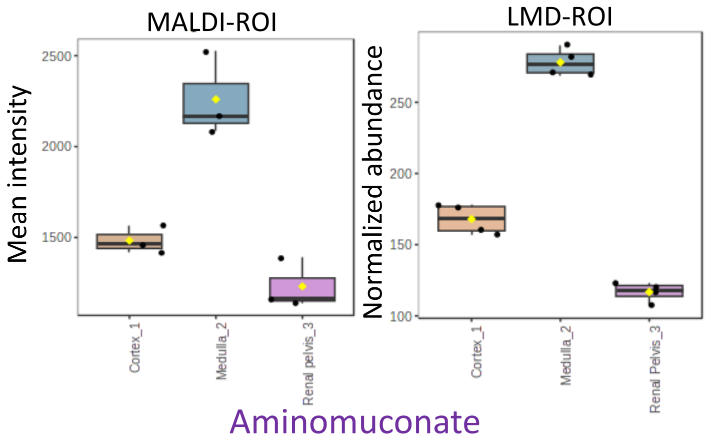

Allantoin

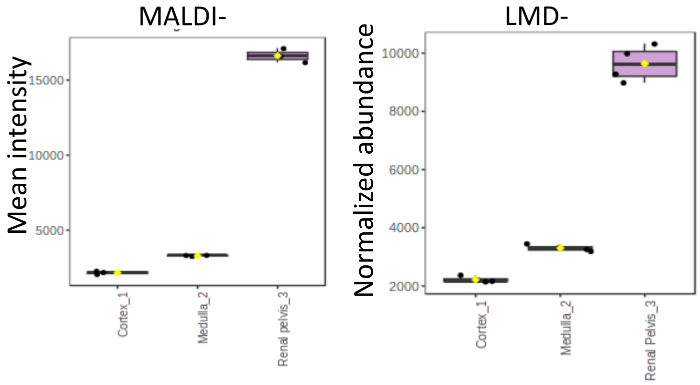

Aminomuconate

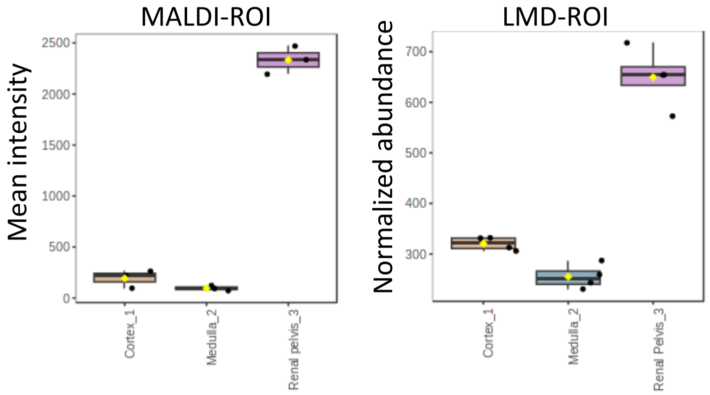

Anserine

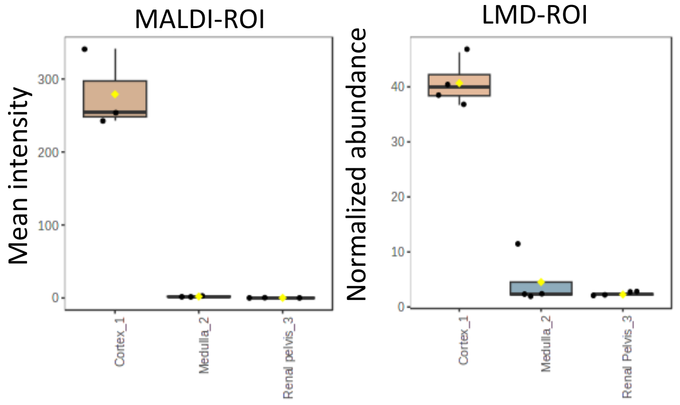

Aspartate

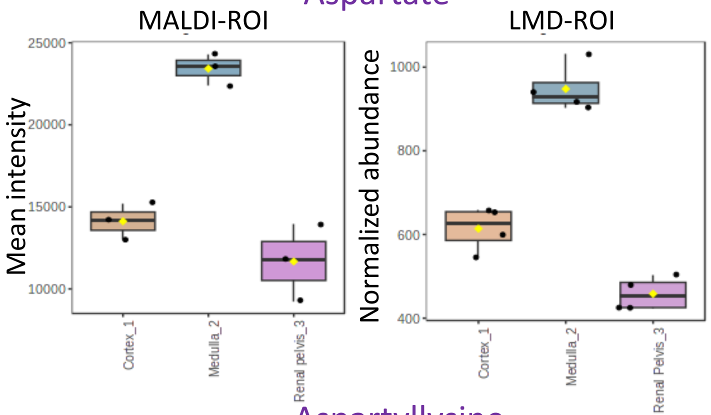

Asparagine

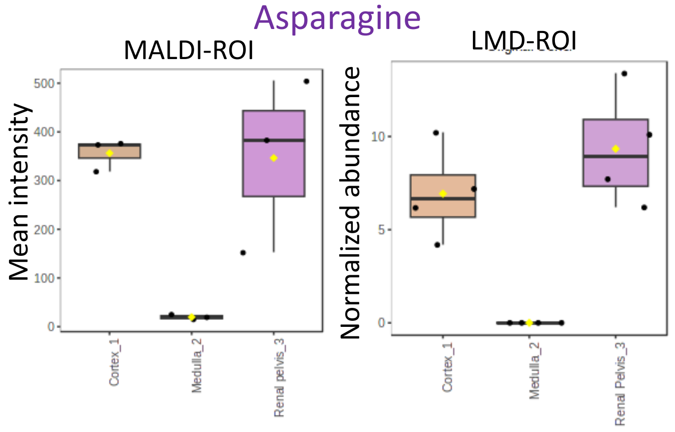

Aspartyllysine

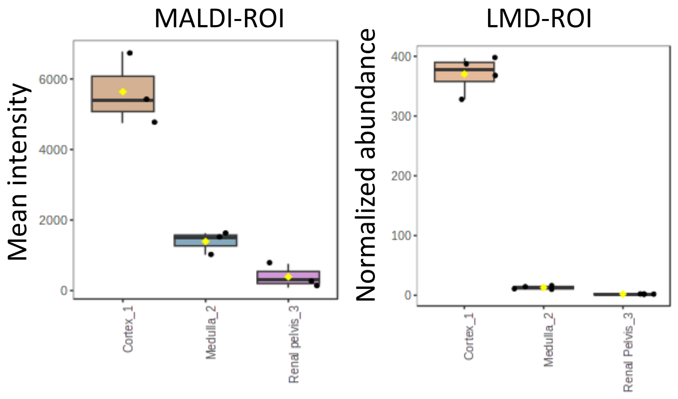

Cystine

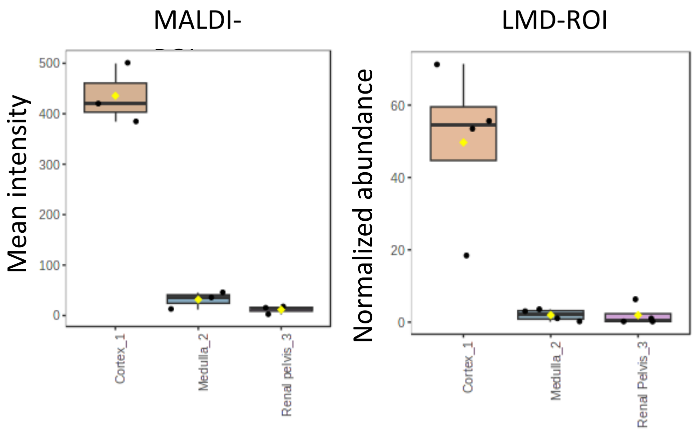

Fumarate

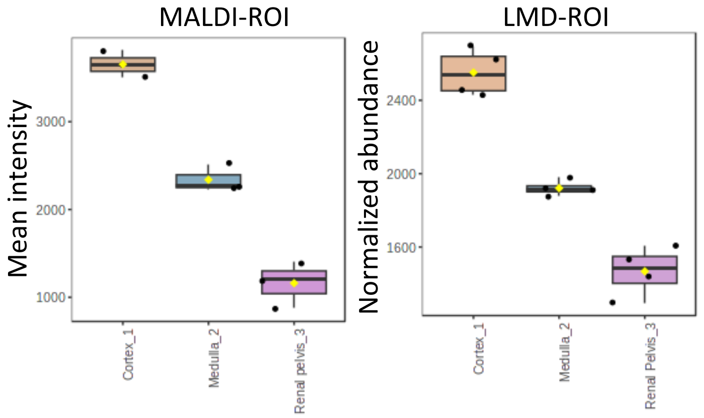

Cytidine

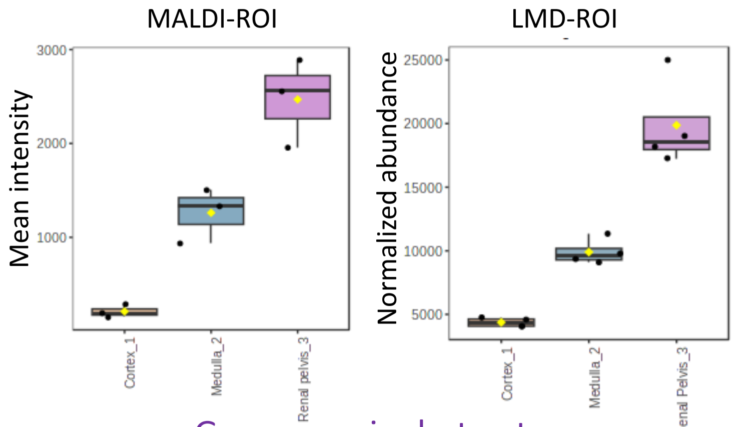

Deoxyinosine

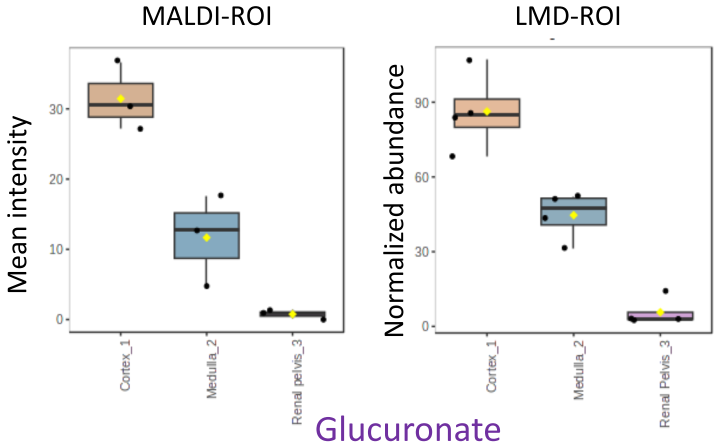

Gamma aminobutyrate

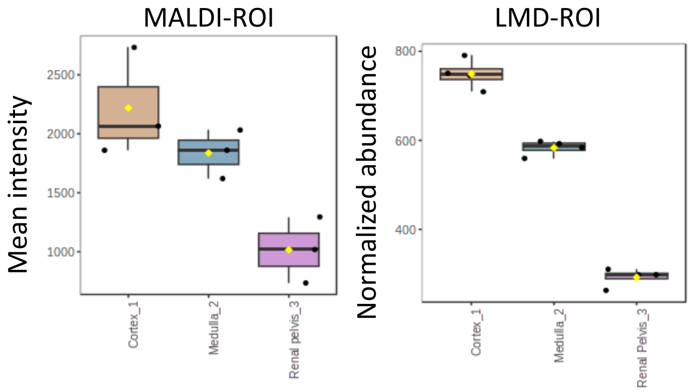

Glucuronate

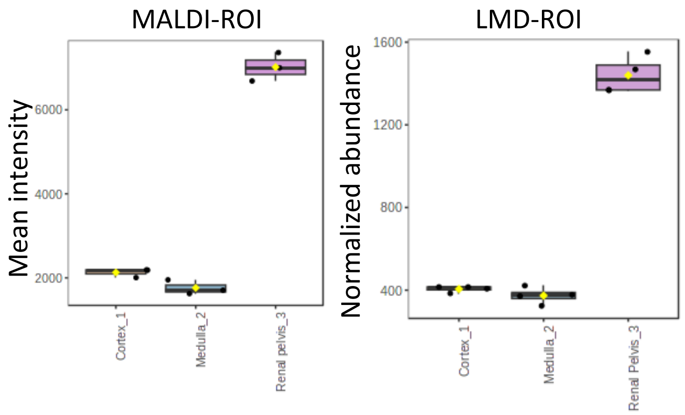

Glucose

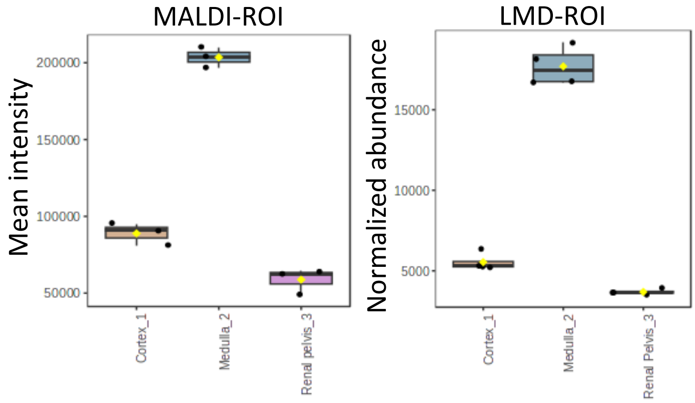

Glutamate

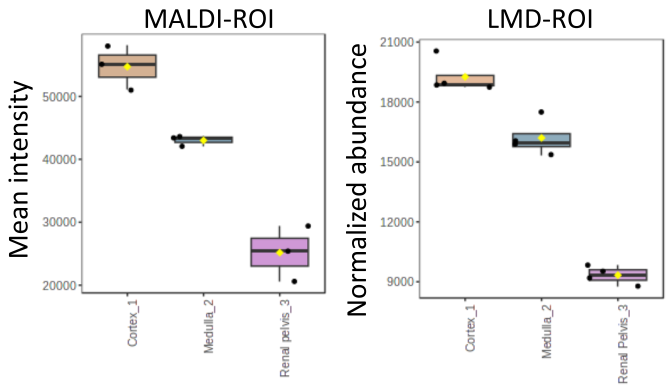

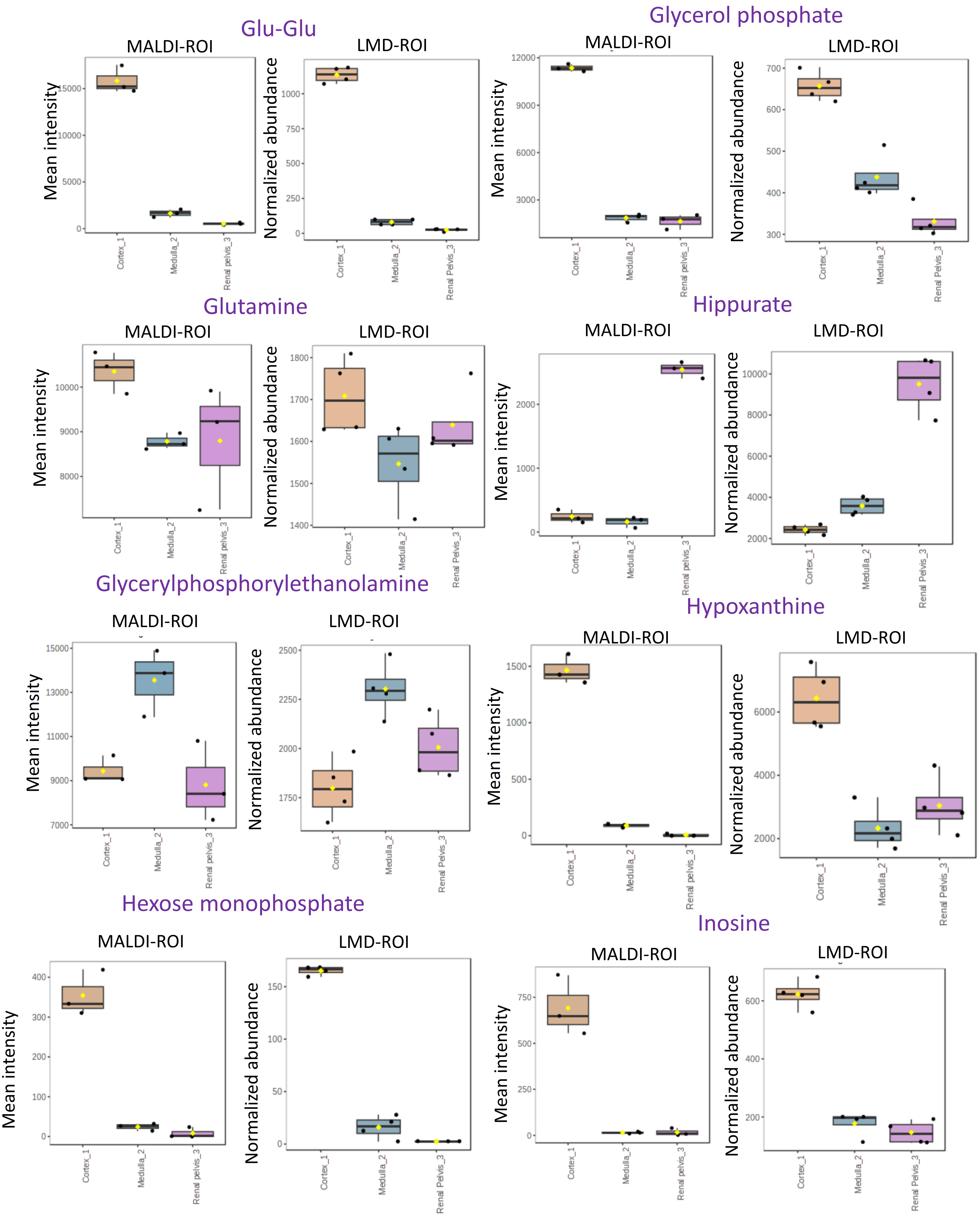

Glu-Glu
MALDI-ROI
LMD-ROI
Glycerol phosphate
MALDI-ROI
LMD-ROI
Glutamine
MALDI-ROI
LMD-ROI
Hippurate
MALDI-ROI
LMD-ROI
Glycerylphosphorylethanolamine
MALDI-ROI
LMD-ROI
Hypoxanthine
MALDI-ROI
LMD-ROI
Hexose monophosphate
MALDI-ROI
LMD-ROI
Inosine
MALDI-ROI
LMD-ROI
Mean intensity
Normalized abundance
Cortex_1
Medulla_2
Renal pelvis_3
Renal Pelvis_3

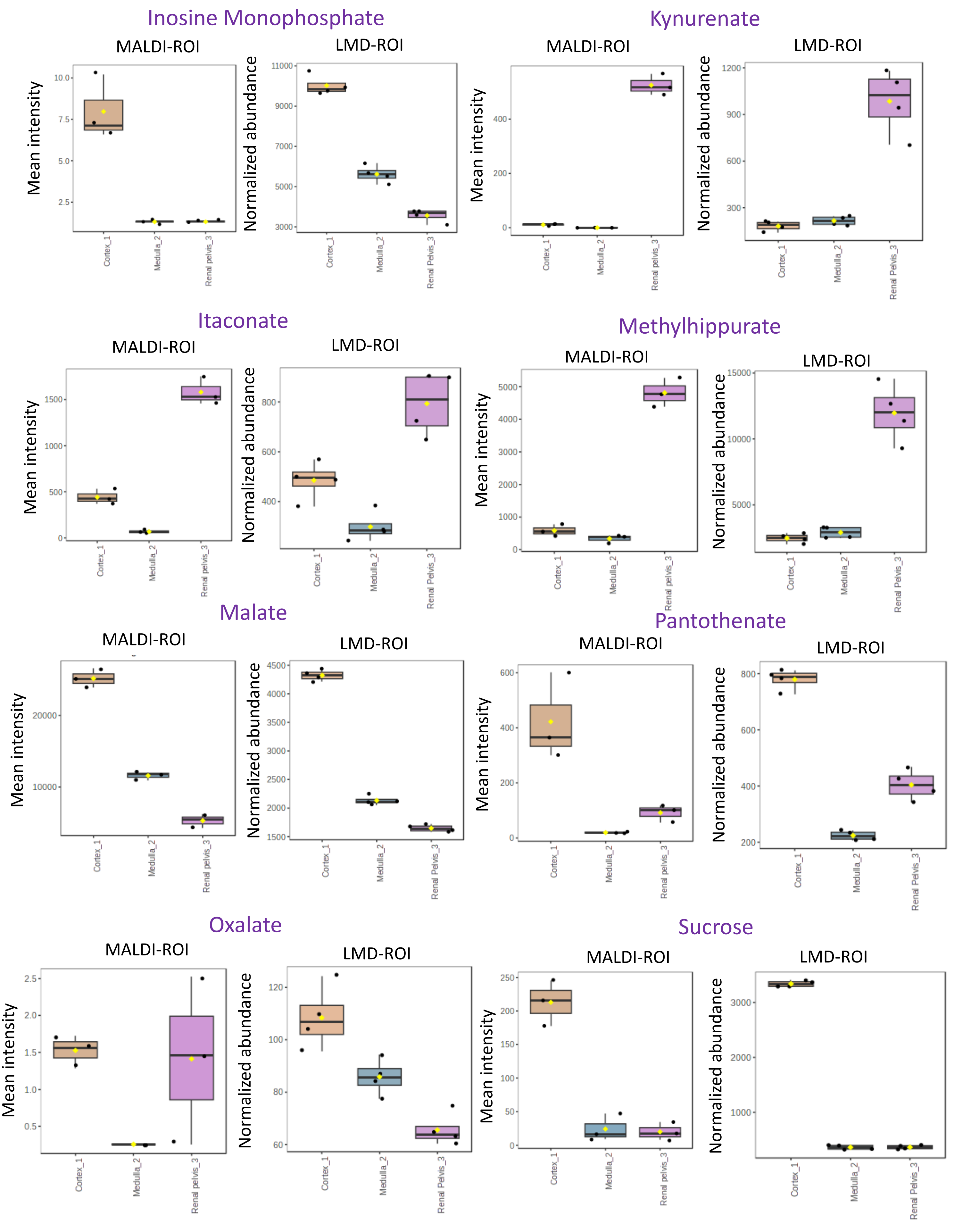

Inosine Monophosphate
MALDI-ROI
LMD-ROI
Mean intensity
Normalized abundance
Cortex_1
Medulla_2
Renal pelvis_3
Renal Pelvis_3
Kynurenate
MALDI-ROI
LMD-ROI
Mean intensity
Normalized abundance
Itaconate
MALDI-ROI
LMD-ROI
Mean intensity
Normalized abundance
Methylhippurate
MALDI-ROI
LMD-ROI
Mean intensity
Normalized abundance
Malate
MALDI-ROI
LMD-ROI
Mean intensity
Normalized abundance
Pantothenate
MALDI-ROI
LMD-ROI
Mean intensity
Normalized abundance
Oxalate
MALDI-ROI
LMD-ROI
Mean intensity
Normalized abundance
Sucrose
MALDI-ROI
LMD-ROI
Mean intensity
Normalized abundance

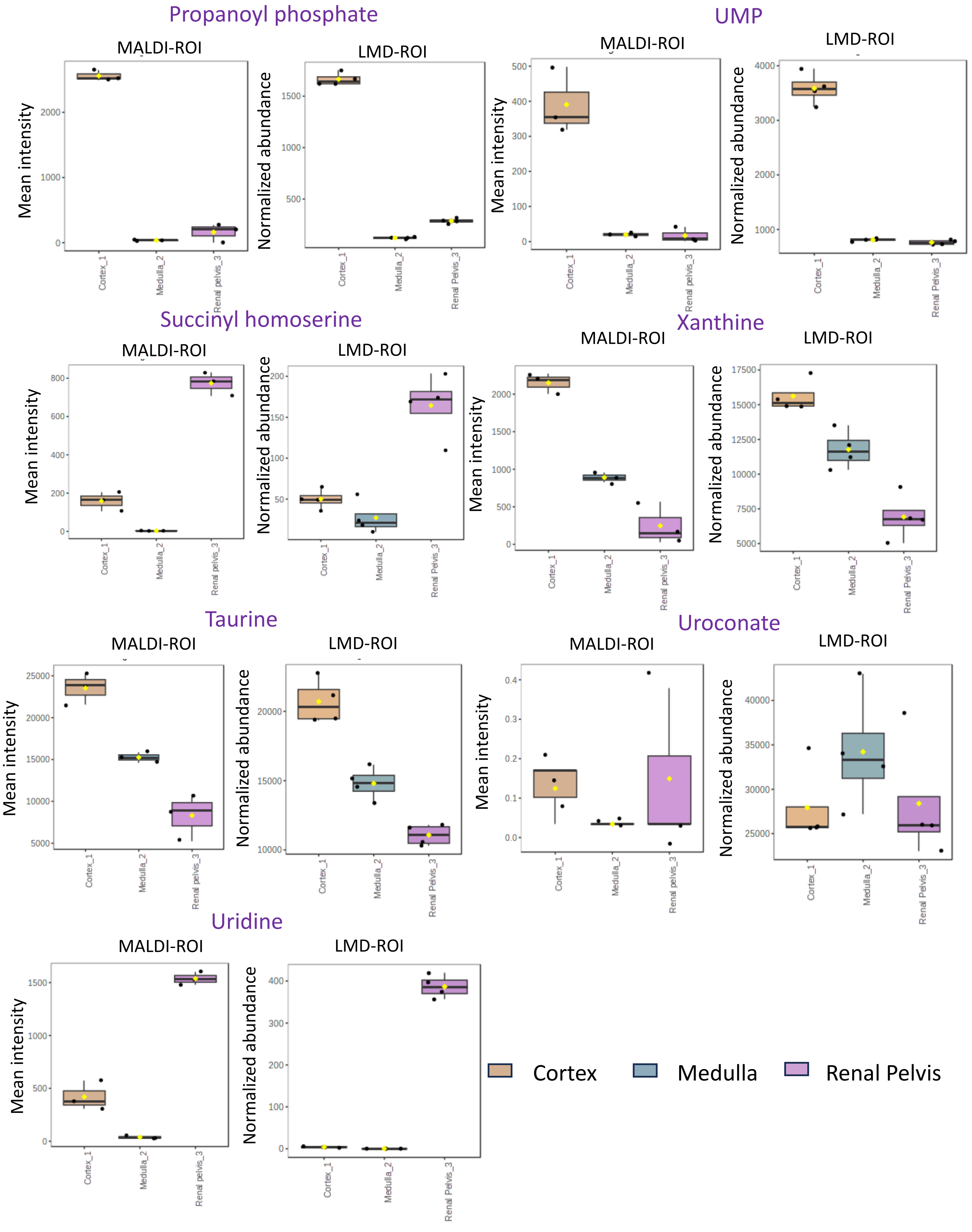

Fig S20. Comparative box plots of commonly identified metabolites from MALDI-MSI and LMD-LC-MS/MS analysis

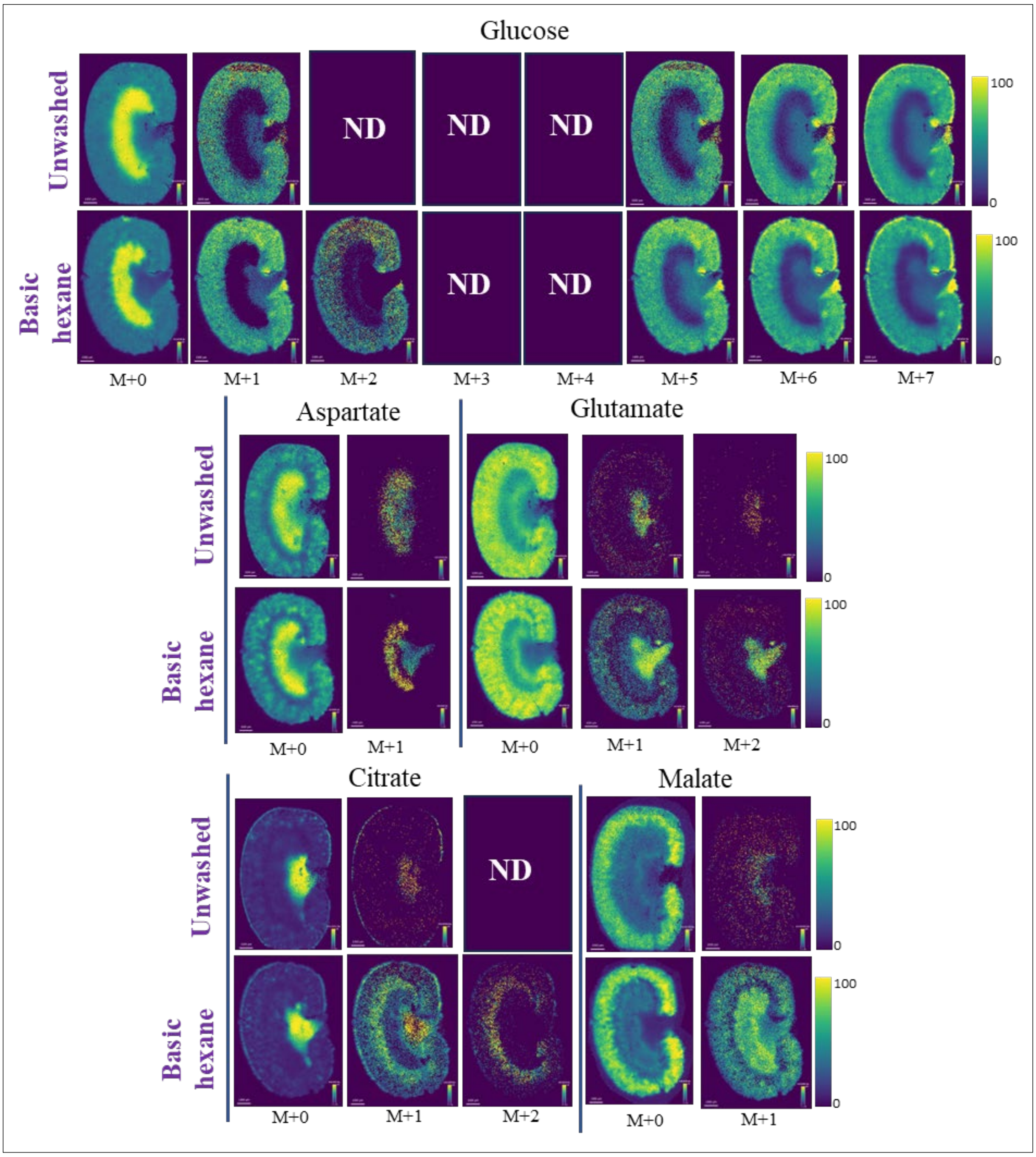

Figure S21. Selected MS ion images of ^{2}H labeled metabolites annotated in mouse kidney tissue demonstrating significant improvement in labeled polar metabolite imaging post basic hexane wash.

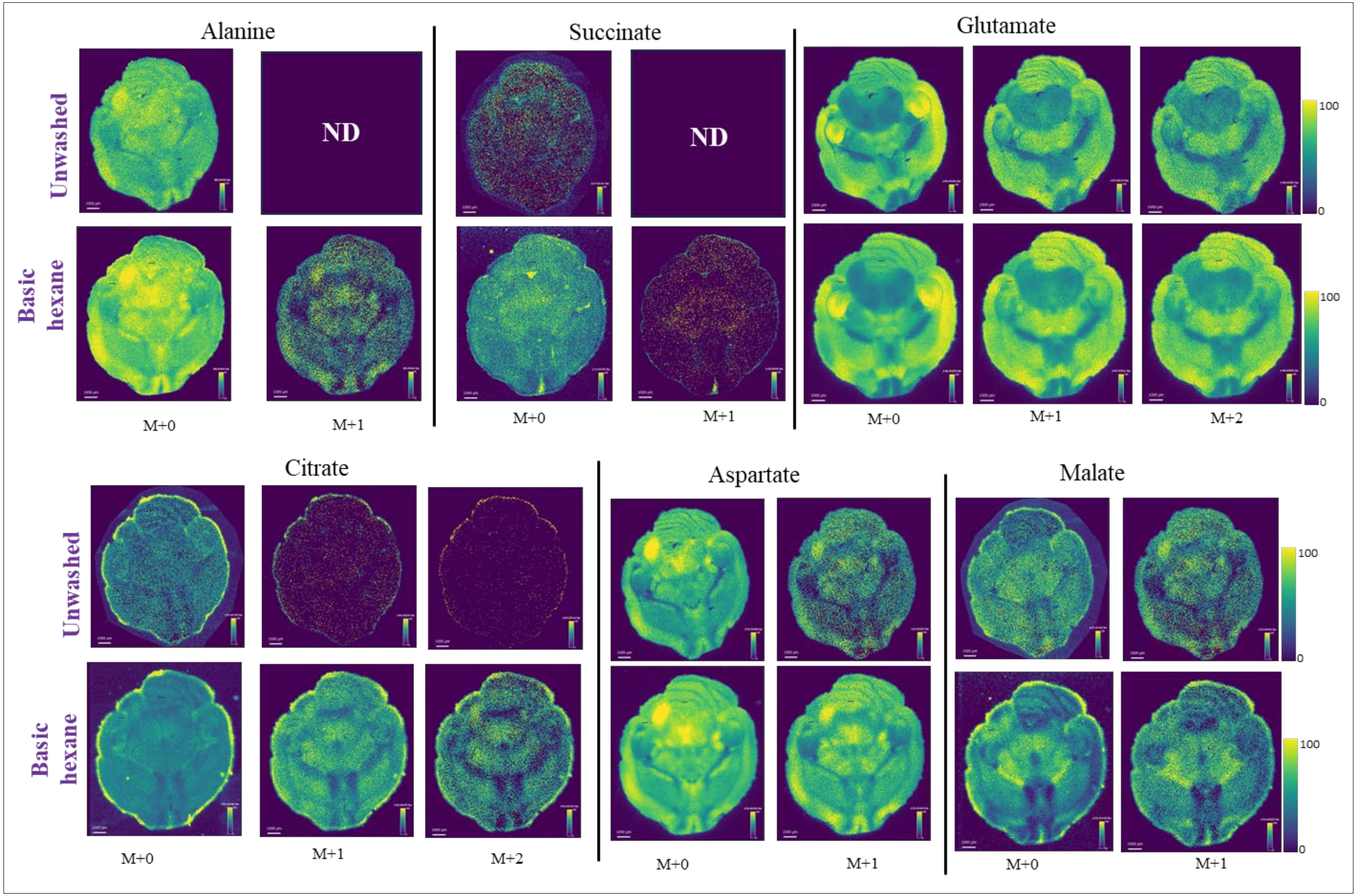

Figure S22. Selected MS ion images of ^{2}H-labeled metabolites annotated in mouse brain tissue showing improved labeled polar metabolite imaging post basic hexane wash.

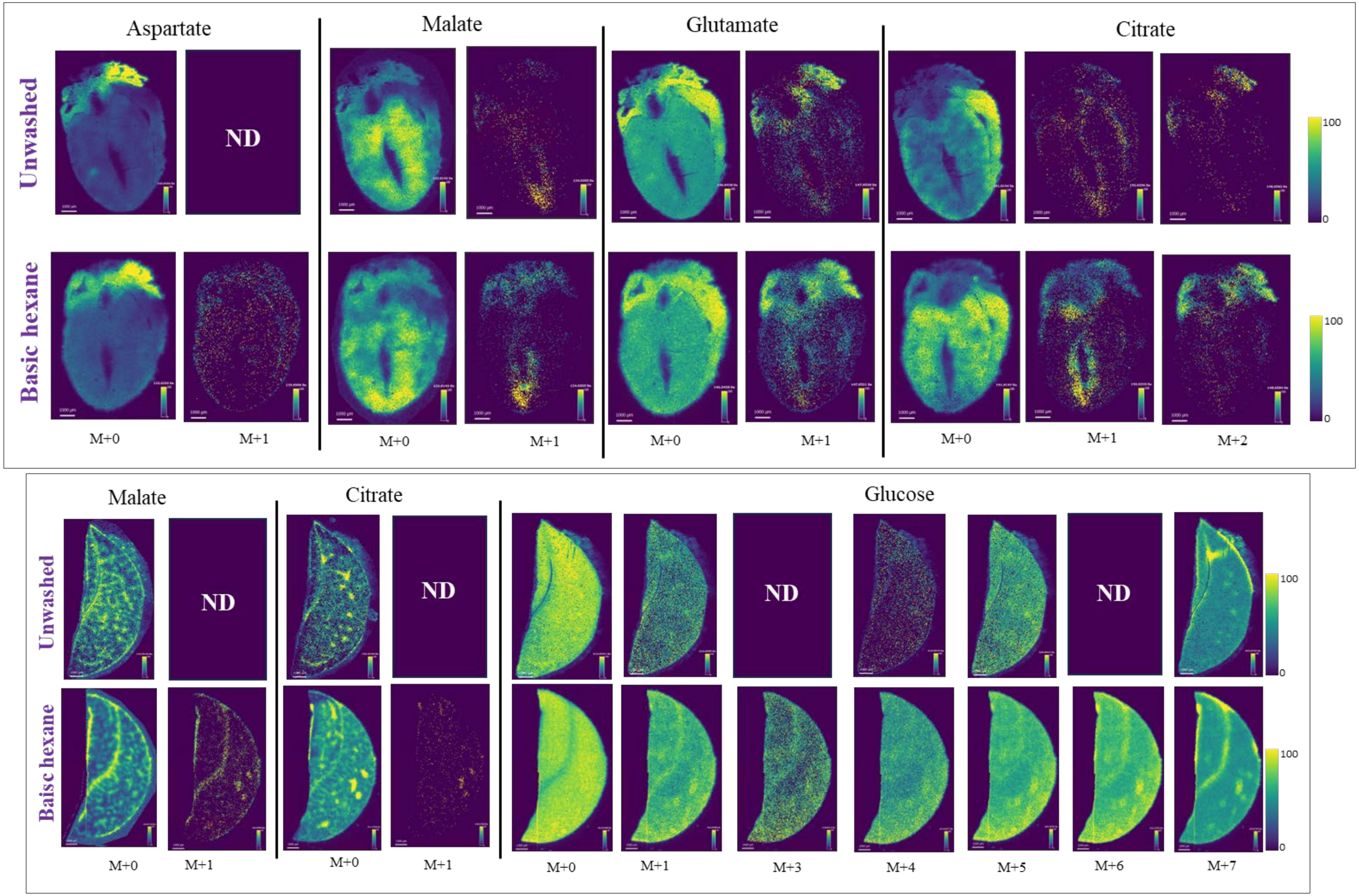

Figure S23. Selected MS ion images of ^{2}H-labeled metabolites annotated in mouse heart and liver tissues showing improved labeled polar metabolite imaging post basic hexane wash.

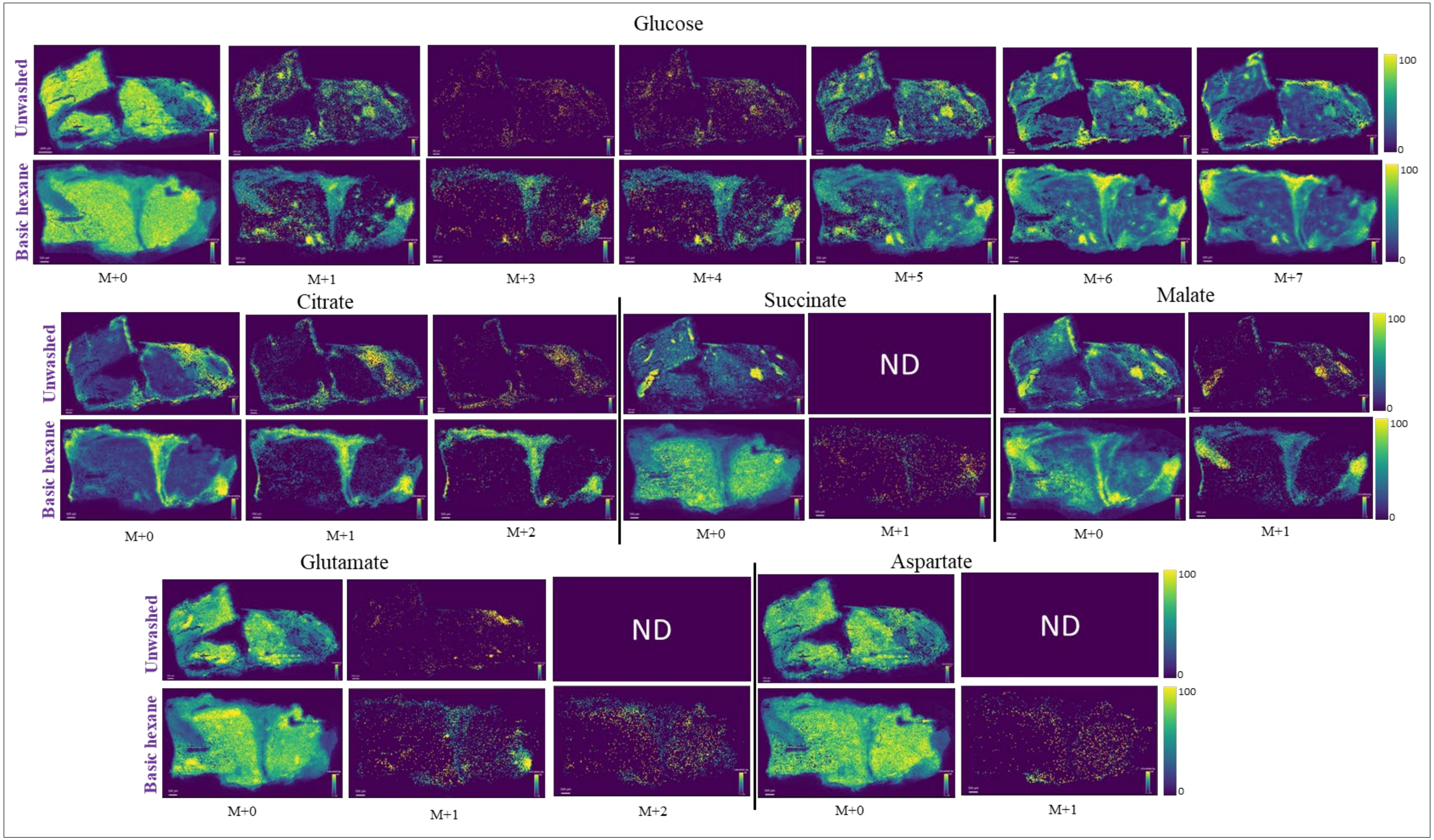

Figure S24. Selected MS ion images of ^{2}H-labeled metabolites annotated in mouse BAT tissue showing improved labeled polar metabolite imaging post basic hexane wash.
