# Supplementary material for: Improved MALDI-MS Imaging of Polar and 2H‑Labeled Metabolites in Mouse Organ Tissues
Source: Anal Chem. 2025 May 13;97(20):10720–8. doi: 10.1021/acs.analchem.5c00620 (PMC12120821; doi:10.1021/acs.analchem.5c00620)
Supplement: Supplementary file 2 [file ac5c00620_si_002.pdf]

Supplementary Information

Supplementary Methods

Improved MALDI-MS Imaging of Polar and ^{2}H-Labeled Metabolites in Mouse Organ Tissues

Siva Swapna Kasarla[1], Antonia Fecke[1], Karl William Smith[1], Vera Flocke[2,3], Ulrich Flögel[2,3], Prasad Phapale[1,2*]

1. Leibniz-Institut für Analytische Wissenschaften—ISAS—e.V., Otto-Hahn-Str. 6b, Dortmund 44227, Germany

2. Experimental Cardiovascular Imaging, Institute for Molecular Cardiology, Heinrich Heine University Düsseldorf, Düsseldorf 40225, Germany

3. Cardiovascular Research Institute Düsseldorf (CARID), Düsseldorf 40225, Germany

4. Department of Environmental Science, Aarhus University, Frederiksborgvej 399, Roskilde, 4000, Denmark

*Corresponding author: prasad.phapale@envs.au.dk

Table of contents

1. Materials--(Page S3)
2. Animal experimentation---(Page S3)
3. Tissue sectioning---(Page S3)
4. Matrix application---(Page S3-S4)
5. MALDI-MS imaging---(Page S4)
6. Hematoxylin and eosin (H &E) staining---(Page S4-S5)
7. MALDI-MS imaging data analysis---(Page S5-S9)
8. Laser capture microdissection (LMD) based micro sampling of tissues---------(Page S9-S10)
9. Tissue sample preparation for LMD metabolomics-----------------------------------(Page S10)
10. LC-MS-MS method for LMD metabolomics--(Page S11)
11. BCA protein content assay---(Page S11)
12. Data analysis for LMD-LC-MS/MS based metabolomics---------------------------(Page S11)
13. Comparison between MALDI-MSI and LMD-LC-MS/MS-------------------------(Page S11)

1. Materials

MALDI matrices such as N-(3-Dimethylaminopropyl)-N′-ethylcarbodiimide hydrochloride (NEDC) and 2,5-Dihydroxybenzoic acid (DHB), HPLC grade solvents including hexane and chloroform were purchased from Sigma Aldrich, Germany. MS grade acetonitrile (ACN) (Cat. No. 0001207802BS), methanol (MeOH) (Cat. No. 0013684102BS) were purchased from Biosolve BV (Valkenswaard, The Netherlands); and were obtained from Merck, Darmstadt, Germany. Ammonium acetate (PCode: 102,326,574, Merck, Germany) and ammonium hydroxide (PCode: 101,344,936, Fluka Analytical, Schwerte, Germany) were used as additives. Synthetic metabolite standards and stable isotope labeled amino acid mixture for mass spectrometry (Product No. 909653) were procured from Merck, Germany. The chromatographic column, Atlantis Premier BEH Z-HILIC (5 µm, 95 Å, 100 × 2.1 mm) was used for metabolite separation and obtained from Waters Corp (Part no. 186,010,000, Milford, MA, USA). The PET membrane slides were purchased from Leica Microsystems, Germany. Ethanol, Mayer's Haematoxylin, Xylene, Eosin Y (0.5% alcoholic solution), and Histomount 290 were purchased from Sigma Aldrich (Germany).

2. Animal experimentation

Eight- to 12-week-old male C57BL/6J mice were obtained from Janvier Labs (Le Genest-Saint-Ile, France) and were housed at the central animal facility of the Heinrich Heine University (Düsseldorf, Germany). Experimental animals (n=3 per group) were kept at 22 ± 1 °C, with unlimited access to water and food and a 12-h day-night rhythm. All animal studies were performed in accordance with the guidelines for the use of laboratory animals under the German Animal Welfare Act. All the experiments were approved by the NRW State Office for Nature, Environment and Consumer Protection (file reference: 81–02.04. 2017.A458). $^{2}H_{7}$-Glucose (2 mg/g body weight) was administrated intraperitoneally as a bolus injection. After 30 min, mice were sacrificed and the liver, kidney, brain, heart, and brown adipose tissue (BAT) were isolated and snap-frozen immediately in liquid nitrogen followed by storage at −80 °C until further use.

3. Tissue sectioning

Tissues (kidney, brain, liver, heart, and BAT) were placed into a Leica CM1860 cryostat for 30 min to equilibrate the tissues to the chamber temperature before sectioning at -20 °C. Distilled water was used to mount the tissue to the sample holder. All the analysed tissues were sectioned at 12 µm thickness at -20 °C except for BAT which was sectioned at -15 °C. The tissue sections were thaw-mounted on Indium tin oxide (ITO) (70-100 ohms, Delta Technologies, USA) glass slides, and stored at -80 °C until further use.

4. Matrix application

Prior to the MALDI-MSI measurements, the sectioned tissue slides were desiccated using a vacuum desiccator for 20 min at -0.09 MPa. The tissue sections were then washed with appropriate solvents as described elsewhere. The washed tissue sections were again desiccated for 20 min as described earlier. NEDC (7mg/ml in 70:25:5 MeOH:ACN:H_2O v/v/v), and DHB (20mg/ml in 90:10 MeOH:H_2O v/v) matrices were freshly prepared and applied using SunCollect MALDI sprayer (SunChrom Wissenschaftliche Geräte GmbH, Friedrichsdorf, Germany).

No. of layers (NEDC)	Flow rate (µL/min)
1-3	5
4-6	10
7-9	15
10-21	20

No. of layers (DHB)	Flow rate (µL/min)
1	10
2	20
3	30
4-12	40

To attain a homogenous distribution of matrices on tissue surfaces, the speed is maintained at 'Low 8' (620 mm/s) for the x-axis and 'Medium 1' (850 mm/s) for the y-axis. Gas pressure was set at 2.5 bar and a z-position of 35 mm for NEDC spraying. Whereas the speed is maintained at 'Low 3' for the x-axis and 'medium 1' for the y-axis. The gas pressure and z-axis are maintained at 2.5 bar and 25 mm respectively for DHB spraying.

5. MALDI-MS imaging

The MALDI-MS imaging experiments were performed on Orbitrap Q-Exactive HF mass spectrometer (Thermo Fisher Scientific GmbH, Bremen, Germany) coupled to an elevated pressure MALDI ion source (Spectroglyph LLC, Kennewick, WA, USA). The 349-nm MALDI Nd:YLF laser (Explorer One, Spectra Physics, Mountain View, CA) was operated at a repetition rate of 500 Hz and pulse energy of 1-2µJ. The laser was focused to a spot size of 20µm. The funnel parameters such as high-pressure funnel (HPF) and low-pressure funnel (LPF) were operated at 98 Vpp and 75 Vpp, respectively, achieved by using 15% RF drive for 720 kHz and 825 kHz respectively. The mass spectrometer was operated in negative- and positive-ion mode in the mass range of *m/z* 50-550 with AGC mode turned off, multi-RF at 12, the mass resolution was set at 120K for *m/z* 200 and a set inject time of 250 ms was used, resulting in 125 shots/pixel and ~3 scans/second. MS instrument calibration was performed with an electrospray ionization source using a Pierce Positive Ion Calibration solution. Whereas, a customized metabolite standard mix including lactate, pyruvate, taurine, glutathione, glucose, and citrate was spiked into Pierce Negative Ion Calibration solution (Thermo Fisher Scientific) to cover the low *m/z* range (*m/z* 50-550). The on-tissue MS-MS was performed in all ion fragmentation-MS/MS (AIF-MS/MS) mode. The isolation width of 1Da was used with varying collision energies ranging from 35-40 eV.

6. Hematoxylin and eosin (H &E) staining

The tissue sections after MSI measurements were stained with H&E by the following steps.

Steps	Procedure	Time	Purpose
1	95% ethanol (EtOH)	30 sec	Removal of MALDI matrix
2	90% EtOH	30 sec	Hydration of tissue
3	80% EtOH	30 sec	Hydration of tissue
4	70% EtOH	30 sec	Hydration of tissue
5	Phosphate buffer saline	5 min	Hydration of tissue
6	Hematoxylin	1 min	Staining step
7	H_2O	10 min	Bluing step (To remove excess Hematoxylin)
8	70% EtOH	30 sec	Preparation step

9	95% EtOH	30 sec	Preparation step
10	Eiosin (0.025% in EtOH)	10 sec	Staining step
11	95% EtOH	30 sec	Removal of excess stain
12	100% EtOH	30 sec	Removal of excess stain
13	Xylol	1 min	Preparation step for storage

After the staining procedure, histomount was used to fix the slides with a coverslip. Stained tissue microscopic images were captured using Brightfield Keyence microscope, model no:VHX-7000N (Keyence Germany GmbH). Serial stitched images were performed at 200x magnification.

7. MALDI MS imaging data analysis

Prior to the MSI data analysis, the raw data was used to calculate the signal-to-noise (S/N) ratios in Qual Browser (v4.6). Briefly, respective peaks were picked from the average spectra. The noise was displayed from the display options. Then, the signal intensity obtained was used to calculate the signal-to-noise ratio in Microsoft Excel.

7a. Downstream data analysis and metabolite annotation using Lipostar

Preliminary images were viewed in ImageInsight (Spectroglyph) and MSI data processing was further performed using LipostarMSI v2.1.0 (Molecular Horizons). Thermo RAW (.raw) and positional files (.xml) were converted to imzML files using the built-in converter for LipostarMSI, which utilises MSconvert from ProteoWizard (3.0.22317) for initial conversion to mzML format. The imzML file was then loaded into LipostarMSI by setting the MSI processing settings as follows:

1. File properties

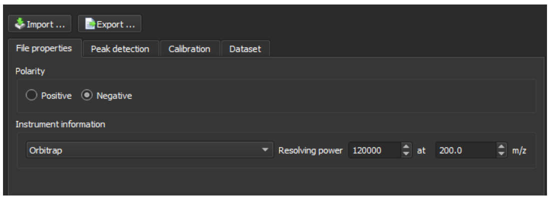

2. Peak detection parameters

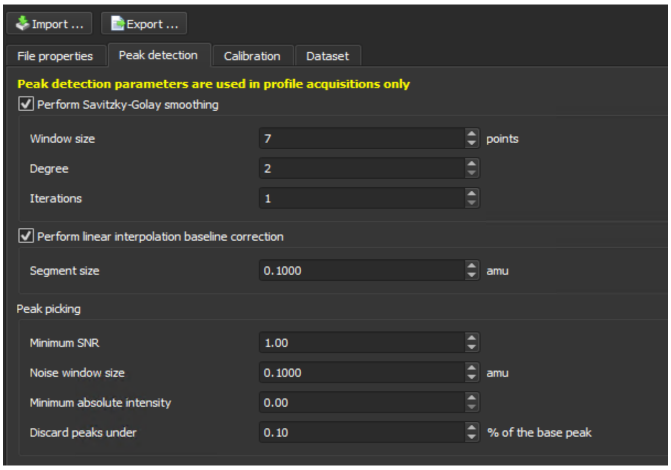

3. Dataset parameters

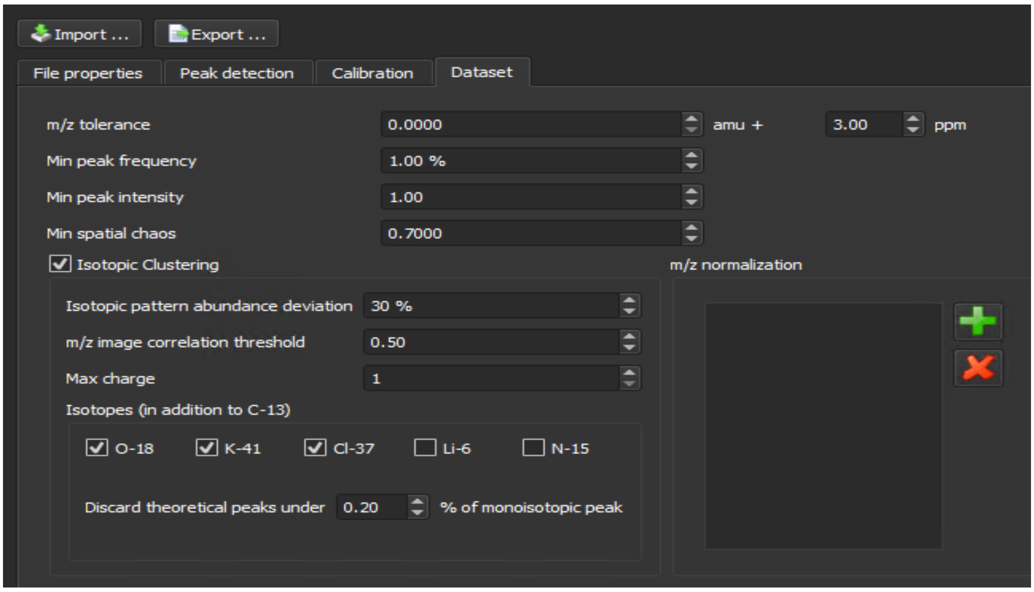

Note: Uncheck the isotope clustering for the labeled datasets.

4. Further data was reviewed by hotspot removal, TIC normalized, and the annotation of metabolites was further validated using METASPACE and confirmed using LC-MS/MS. The log base 2 intensities on-tissue region for all the wash conditions were extracted among five tissues for further comparisons.
5. The fold change values of before wash (unwashed) with respect to after wash (hexane or chloroform or basic hexane) were calculated in Mircosoft Excel. To enable comparison across variables with different scales, the obtained fold change values were normalized using min-max scaling to a range of 0–10. The linear transformation preserved the relative distribution of the data and facilitated comparison across different samples. The obtained fold change values are scaled to 0-10 followed by heatmap construction using "R Programming".

7b. **MALDI-ROI analysis**

We used ShinyCardinal (v3.4), a web application tool for image visualization, segmentation and statistical analysis of ROI's as described below.

The converted .imzML files from the previous step are used for further statistical analysis in ShinyCardinal. The web version of ShinyCardinal was installed by following the user instructions (https://sourceforge.net/projects/shinycardinal/).

Step:1 Read MSI data

1. To read the MSI files, we have chosen the method 2 i.e., "Read uploaded MSI files" by uploading both .imzML and ibd files to the server
2. The parameters used in choose MSI data type are as follows (see highlighted ones)

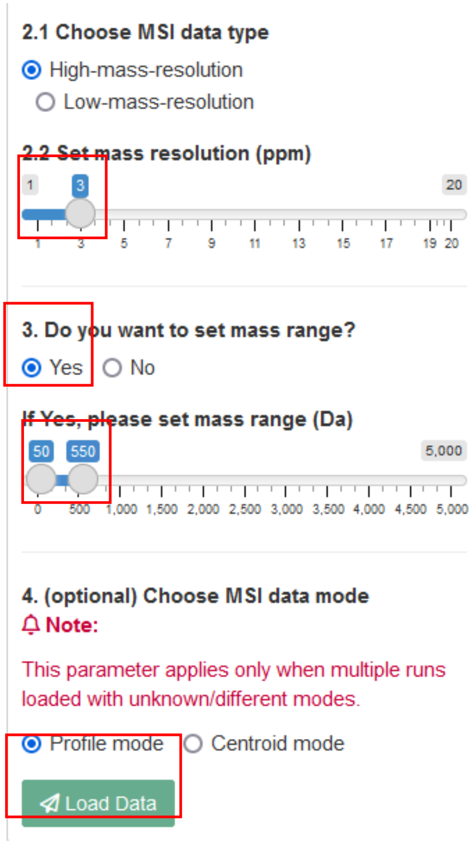

3. Then the mean spectra and calculating the reference peaks were performed in the “Calculate Mean Spectra” and “Calculate Reference Peaks” tab respectively by the following parameters:

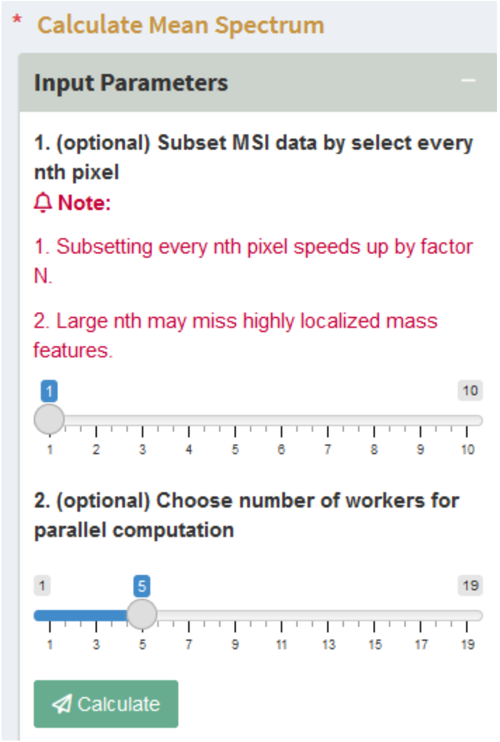

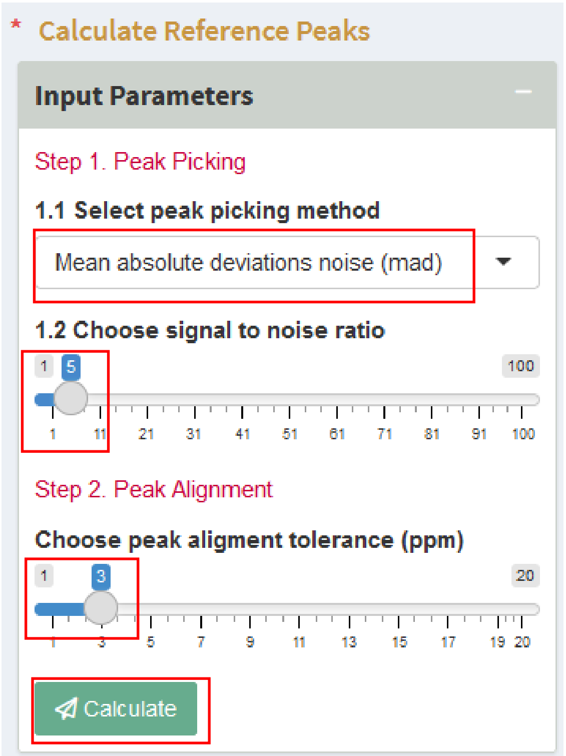

4. The MSI data was further processed in the "Process MSI data" tab and the .rds file was downloaded. The parameters used are as follows:

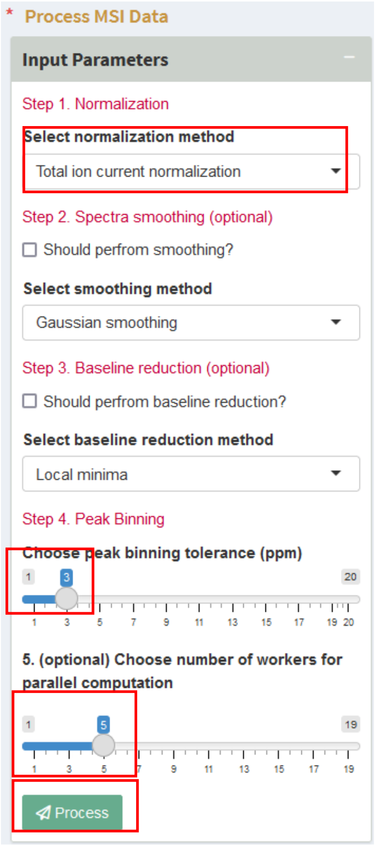

Step 2: ROI analysis

1. Initially to differentiate the regions of regions, metabolites distributed specifically are selected and the masses such as 215.0327 [Glucose +Cl]$^{-}$; 173.0092 [Aconitate-H]$^{-}$; 133.0143 [Malate-H]$^{-}$; The mass overlay function is used to visualize the differential regions.
2. ROI analysis was performed in "Image Visualization" module. In the "Image Analysis" tab draw the ROI by clicking on "new ROI". This module requires specific ROI name format for statistics. Use roiName_metaName format to define ROI and meta data. For example: ROI1_Cortex; ROI2_Cortex; ROI1_Medulla; ROI2_Medulla. As the hypothesis test requires replicates, we have divided sample area into sub-ROIs.
3. The ROI's are analysed by clicking on Compare ROI's. After the analysis is finished, the data containing mean intensities of the masses from the selected ROI's, foldchanges were exported to csv and further selective masses are isolated followed by statistical analysis.

8. Laser capture microdissection (LMD) based micro sampling of tissues

The consecutive liver and kidney tissue sections (n=4 technical replicates) were thaw mounted on polyethylene naphthalate (PEN) membrane slides at -20°C and stored at -80°C until further use. The mounted tissue samples were drying under the vacuum for about 20min before use. The basic hexane wash strategy was applied to the tissues mounted on PEN membrane slides

pre LMD. The regions of interests (ROI) were extracted using Leica LMD6000 (Germany). The defined cortex, medulla, renal pelvis regions (n=4 technical replicates) were identified based on the H&E staining and MALDI-ROI analysis. To define the region of interests, H&E slide was placed on one position of the slide holder and the serially sectioned slide mounted on PEN membrane slide was placed on other position of the slider holder. The microscopy was operated at "Basic control" mode, using "transmitted light- brightfield (TL-BF)" contrast methods at a 5x magnification. The microscope camera was set to "DFC365 FX" in the camera settings for better contrast differentiation and visualization. Laser parameters such as laser power is maintained at 52%, aperture at 20%, speed 12sec. Initially, the regions are drawn by simply selecting the position of the H&E tissue slide on the slider holder. By using "Draw and Cut option" the defined kidney regions were drawn. After defining and drawing the ROIs on H&E tissue, the drawn shapes are then transferred on to the PEN membrane tissue slide. The defined areas are extracted from the PEN membrane slide by clicking on start cut. An area of around 0.8 mm^2 of cortex, medulla and renal pelvis is extracted and collected in to 0.5 ml Eppendorf caps and caps are closed carefully without losing the tissue. Likewise, the liver tissue mounted on PEN membrane slide are washed with basic hexane as mentioned earlier. Later, 1 mm^2 tissue area was excised from the washed tissue and unwashed tissue. Then the tubes are spined for 20 sec which facilitates the samples to settle at the bottom. The spined samples were stored at -80 until further processing.

9. Tissue sample preparation for LMD-LC-MS/MS metabolomics

The tuned metabolomics method for low input samples is as follows, metabolites were extracted by organic solvent-based protein precipitation method. The 150 µL of ice-cold dilution solvent which contains 40:40:20 ACN: MeOH: H2O (v/v) was added to the 0.5ml LMD eppendrof tubes which contains excised tissue samples. After addition of the solvent, the sample is transferred into the 2mL tubes to ease further metabolite extraction procedure. Subsequently, around 10-15 extraction beads (Cat No. C20000021) for tissue disruption were introduced into each tube containing a tissue sample, and the mixture was homogenized using a Bioruptor (Diagenode Inc, Germany). The parameters of bioruptor such as sonication cycle of 30 sec ON/ 30 sec OFF with total sonication time of 5 cycles at 4 °C to facilitate the extraction of metabolites. After homogenization, the extraction solvent is transferred into new eppendorfs and 8 µL of internal standard (IS) containing isotope-labeled amino acid mixture (25nM) was added to all the tissue samples. The samples were then vortexed at 4 °C for 20 min and stored at −80 °C overnight for protein precipitation followed by centrifugation at 15,000 rpm for 20 min at 4 °C. After centrifugation, the protein pellet was processed for BCA protein estimation assay followed by proteomic analysis. The aqueous supernatant was collected into a separate microcentrifuge tube without disturbing the pellet and dried under nitrogen evaporator at room temperature for 30 min. The samples were then stored at −80 °C until further analysis. Prior the LC-MS data analysis, the dried extracts were reconstituted with 40 µL of 70:30 % ACN: H2O (v/v) buffer. The reconstituted samples were then vortexed vigorously for 15 min, followed by a 2-hour incubation at −80 °C. Thereafter, samples were centrifuged at 15,000 rpm for 10 min at 4 °C, and the resulting supernatant was collected and transferred into fresh LC-MS vials for subsequent data acquisition.

10. LC-MS/MS method for LMD based metabolomics

LC-MS measurements were performed on the Vanquish Duo UHPLC-system (Thermo Fisher Scientific, Waltham, MA, USA) equipped with a dual pump, an autosampler, and a thermostatic column compartment with the Atlantis Premier BEH Z-HILIC column. The mobile phases used were 100 % water with 10 mM ammonium acetate and 0.1 % ammonium hydroxide, pH 8.5 as a mobile phase A and 100 % ACN as a mobile phase B with a flow rate of 0.4 mL/min. The elution gradient conditions were as follows: 95 % B, 0–2 min; 95–80 % B, 2–7.7 min; 80–70 % B, 7.7–9.5 min; 70–10 % B, 9.5–12 min; 10–30 % B, 12–16 min; 30–95% B 16.5–19 min. The mass spectrometer used for this study was Orbitrap Fusion Lumos Tribrid Mass Spectrometer (Thermo Fisher Scientific, Waltham, MA, USA) with heated electrospray ionization (HESI) source. Data was acquired in DDA mode in negative-ion mode of metabolites at a resolution of 120,000 within the mass range of 65–850 m/z. For the DDA scans, the system was configured to fragment the ten most intense ions (Top10). The HESI source was operated at a voltage of 3000 V in negative-ion mode. Source parameters such as sheath gas and auxiliary gas were set to 35 arbs and 10 arbs respectively. The ion transfer tube was heated to 300 °C; the vaporizer temperature was set to 310 °C. The orbitrap was set at a resolution of 15,000 with a HCD stepped collision energy of 20,35,45 %.

11. BCA protein content assay

The BCA standards were prepared at a working range of 1-250 µg/mL. The protein pellet collected from the LMD-metabolomics samples were resuspended in 150 µL of lysis buffer (SDS, protease inhibitor, PhosSTOP™, NaCl, Tris HCL, ammonium bicarbonate). To disrupt the pellet, all the samples are sonicated using ultrasonicate lancer at a power of 50 W for 5 sec. Then 25 µL of samples is used for BCA assay and the rest of the lysate is used for proteomic analysis. The BCA protein assay is performed in duplicates and the absorbance was taken as 562 nm. The protein concentrations obtained were used for normalizing the metabolomics data.

12. Data analysis for LMD-LC-MS/MS based metabolomics

Thermo Xcalibur Qual Browser and Progenesis QI (v.3.0) software were used for initial data evaluation, calculate% CVs for spiked IS, and pooled QC evaluation with PCA score plot. The untargeted workflow in Progenesis QI is as follows: peak alignment, feature detection, peak picking, deconvolution and elemental composition prediction. Metascope plug of Progenesis QI was used for identification of metabolites by using in-house library of ~500 metabolites with accurate mass, and a retention time cut-off of 0.5 min for the database search. The online spectral libraries were also used for further confirmation. Additionally, all detected peaks were verified and validated manually.

13. Comparison between MALDI-MSI and LMD-LC-MS/MS

The metabolite intensities of the defined ROI's from the MALDI-MSI and LMD-LC-MS/MS were extracted from the ShinyCardinal v3.4 and Progenesis QI respectively. The statistical analysis of the metabolite intensities acquired from both the techniques were analysed in Metaboanlyst 6.0 v separately. The heatmaps of the differential metabolites was constructed based on the ANOVA p-value of <0.05. The bar garphs were extracted from the volcano plot analysis. The parameters such as fold change (FC) was kept that >1.5 and p-value of <0.05 were used to find the significant features based on either biological significance, statistical significance.
